# Scientific evidence of sodium-glucose cotransporter-2 inhibitors for heart failure with preserved ejection fraction: an umbrella review of systematic reviews and meta-analyses

**DOI:** 10.3389/fcvm.2023.1143658

**Published:** 2023-05-12

**Authors:** Runmin Li, Guohua Dai, Hui Guan, Wulin Gao, Lili Ren, Xingmeng Wang, Huiwen Qu

**Affiliations:** ^1^ First Clinical Medical College, Shandong University of Traditional Chinese Medicine, Jinan, China; ^2^ Department of Geriatrics, Affiliated Hospital of Shandong University of Traditional Chinese Medicine, Jinan, China; ^3^ College of Traditional Chinese Medicine, Shandong University of Traditional Chinese Medicine, Jinan, China

**Keywords:** sodium-glucose cotransporter-2 inhibitor, heart failure with preserved ejection fraction, umbrella review, overview, systematic review, meta-analysis, evidence quality assessment

## Abstract

**Background:**

It remains controversial whether sodium-glucose cotransporter-2 inhibitors (SGLT-2is) are effective in treating heart failure with preserved ejection fraction (HFpEF).

**Purpose:**

The objective of this umbrella review is to provide a summary of the available evidence regarding the efficacy and safety of SGLT-2is for the treatment of HFpEF.

**Methods:**

We extracted pertinent systematic reviews and meta-analyses (SRs/MAs) from PubMed, EMBASE, and the Cochrane Library that were published between the inception of the database and December 31, 2022. Two independent investigators assessed the methodological quality, risk of bias, report quality, and evidence quality of the included SRs/MAs in randomized controlled trials (RCTs). We further evaluated the overlap of the included RCTs by calculating the corrected covered area (CCA) and assessed the reliability of the effect size by performing excess significance tests. Additionally, the effect sizes of the outcomes were repooled to obtain objective and updated conclusions. Egger's test and sensitivity analysis were used to clarify the stability and reliability of the updated conclusion.

**Results:**

This umbrella review included 15 SRs/MAs, and their methodological quality, risk of bias, report quality, and evidence quality were unsatisfactory. The total CCA for 15 SRs/MAs was 23.53%, indicating a very high level of overlap. The excess significance tests did not reveal any significant results. Our updated MA demonstrated that the incidence of the composite of hospitalization for heart failure (HHF) or cardiovascular death (CVD), first HHF, total HHF, and adverse events as well as the Kansas City Cardiomyopathy Questionnaire Total Symptom Score (KCCQ-TSS) and 6 min-walk distance (6MWD) were all substantially improved in the SGLT-2i intervention group compared to the control group. However, there was limited evidence that SGLT-2is could improve CVD, all-cause death, plasma B-type natriuretic peptide (BNP) level, or plasma N-terminal pro-B-type natriuretic peptide (NT-proBNP) level. Egger's test and sensitivity analysis proved that the conclusion was stable and reliable.

**Conclusions:**

SGLT-2 is a potential treatment for HFpEF with favourable safety. Given the dubious methodological quality, reporting quality, evidence quality, and high risk of bias for certain included SRs/MAs, this conclusion must be drawn with caution.

**Systematic Review Registration:**

https://inplasy.com/, doi: 10.37766/inplasy2022.12.0083, identifier INPLASY2022120083.

## Introduction

Heart failure with preserved ejection fraction (HFpEF), as measured by left ventricular ejection fraction (LVEF), is observed in approximately 50% of all patients with heart failure and is regarded as a significant subtype (1, 2). HFpEF is more common in females and the elderly. The incidence of HFpEF increases with age, and the proportion of females is higher than that of males in all age groups (3–5). In addition to the high prevalence, HFpEF is related to a significant decline in quality of life (6), and both the mortality risk and hospital readmission rates of HFpEF are higher than those of heart failure with reduced ejection fraction (HFrEF) (7–9). The combination of these two elements makes HFpEF a serious public health concern and places a significant burden on society and families (10, 11). However, as a heterogeneous disease, the complex pathogenic factors and various pathophysiological characteristics of HFpEF present challenges to the formulation of treatment (12–14). Although advancements have been reported for certain phenotypes of patients with HFpEF, unequivocal class Ia and Ib obligatory prescription recommendations to decrease mortality and morbidity in patients with HFpEF have not been reported according to the 2021 ESC guidelines and 2022 AHA/ACC/HFSA guidelines (1, 15).

Sodium-glucose cotransporter-2 inhibitors (SGLT-2is) are a new family of oral hypoglycaemic drugs that decrease serum glucose by inhibiting glucose reabsorption by proximal renal tubules and enhancing urine glucose excretion (16). Multiple randomized controlled trials (RCTs) have indicated that SGLT-2is have cardioprotective and renoprotective effects regardless of hyperglycaemia and decrease the incidence of hospitalization for heart failure (HHF) and cardiovascular death (CVD) in patients with HFrEF (17–19). As a result, SGLT-2is are recommended as the foundation for HFrEF therapy (1, 15). Nevertheless, the benefits of SGLT-2is in treating HFpEF remain controversial, and the results of several large-scale RCTs have been inconsistent. For example, the EMPEROR-Preserved trial (20–22) found that SGLT-2is reduced the risk of first HHF and the composite of HHF or CVD in patients with HFpEF, whereas the VERTIS-CV trial (23) showed opposite outcomes. Similar trends were observed for plasma N-terminal pro-B-type natriuretic peptide (NT-proBNP) level and 6 min-walk distance (6MWD). SGLT-2is did not improve the value of 6WMD in the EMPERIAL-Preserved trial (24), which was opposite in the PRESERVED-HF trial (25). The EMPEROR-Preserved trial (20–22) found that SGLT-2is helped to reduce NT-ProBNP levels in patients with HFpEF, whereas the PRESERVED-HF trial (25) showed the opposite outcomes. However, this situation has drastically changed with the recent publications of several cases of large-scale RCTs. For instance, the DELIVER trial, an international, multicenter, double-blind, randomized, placebo-controlled trial done in 350 healthcare centers and hospitals across 20 countries, has shown that SGLT-2is could reduce the risk of first HHF and the composite of HHF or CVD in patients with HFpEF but has no significant improvement in reducing the incidence of CVD and all-cause death (26–28). Notably, based on the result of the “Empagliflozin Outcome Trial in Patients with Chronic Heart Failure with Preserved Ejection Fraction”, the 2022 AHA/ACC/HFSA guidelines have assigned a recommended grade of II to SGLT-2is, which has attracted a considerable amount of attention from researchers, clinicians and patients (20).

As a result of the growing attention to SGLT-2is for the treatment of HFpEF, researchers have performed systematic reviews and meta-analyses (SRs/MAs) to assess the therapeutic benefits. By appropriately adhering to the relevant research guidelines, the SRs/MAs provide reliable medical evidence (29). Unfortunately, the strength of the conclusions is diminished to some degree by the existing lack of methodological quality assessment of SRs/MAs related to SGLT-2is for the treatment of HFpEF. Umbrella reviews offer a novel approach to combining SRs/MAs by assessing their methodological quality and reestimating outcomes, which may offer high-quality evidence for clinical decision-making. Consequently, the purpose of this research is to combine existing evidence, assess the quality of prior SRs/MAs pertaining to the efficacy of SGLT-2is in treating patients with HFpEF, and recalculate the effect size by an umbrella review.

## Methods

This umbrella review of SRs/MAs follows the guidelines outlined by the Cochrane Handbook (30) and other high-quality umbrella reviews (31, 32). We registered a protocol for this study on the INPLASY platform (DOI: 10.37766/inplasy2022.12.0083; Registration Number: INPLASY2022120083).

### Data sources and search strategy

PubMed, EMBASE and the Cochrane Library were searched for relevant studies. Searches were conducted by two study investigators (RM-L and H-G) independently from inception to December 31, 2022. We combined keyword search with free word search as strategy, and the keywords included “Meta-Analysis as Topic”, “Systematic review”, “Sodium-Glucose Transporter 2 Inhibitors”, and “Heart Failure, Preserved Ejection Fraction”. Based on this, both the websites for study registration (ClinicalTrials.gov) and the reference lists of included SRs/MAs were manually examined to identify additional relevant studies for this umbrella review. There were no restrictions placed on language use or region of publication. All the detailed strategies were shown in Supplementary Table S1.

### Inclusion and exclusion criteria

The following were the criteria for inclusion: (a) study design: this umbrella review included publicly published SRs/MAs based on RCTs concerning the efficacy of SGLT-2is in treating patients with HFpEF; (b) population: patients with HFpEF were defined based on the ESC guidelines (1), AHA/ACC/HFSA guidelines (15) or Chinese guidelines for the diagnosis and treatment of heart failure 2018 (33) with an LVEF of ≥40% or 50%; (c) intervention and comparison: the intervention group was given SGLT-2is, while the control group was given placebo or conventional treatment (CT); (d) outcomes: main outcomes included first or total HHF, CVD, all-cause death, and the composite of HHF and CVD. Based on this, additional outcomes included plasma B-type natriuretic peptide (BNP) level, NT-proBNP level, change of NT-proBNP, 6MWD, the Kansas City Cardiomyopathy Questionnaire Scores, including the Kansas City Cardiomyopathy Questionnaire Total Symptom Score (KCCQ-TSS), the Kansas City Cardiomyopathy Questionnaire Physical Limitation (KCCQ-PL), the Kansas City Cardiomyopathy Questionnaire Clinical Summary Score (KCCQ-CSS), and the Kansas City Cardiomyopathy Questionnaire Overall Summary Score (KCCQ-OSS), the ratio of early mitral inflow velocity to mitral annular early diastolic velocity (*E*/*e*′) and adverse events (hypoglycaemia, diabetic ketoacidosis, renal events, urinary infection, and any unfavourable or unintended signs, symptoms, or disease, including abnormal laboratory values).

The following were the criteria for exclusion: (a) cell or animal-based studies; (b) study protocols, conference abstracts, editorials, case reports, letters, narrative reviews, and umbrella reviews; and (c) unavailability of data required for this umbrella review.

### Literature screening and data extraction

Identified articles were imported into EndNote X9, and duplicates were eliminated. Two study investigators (WL-G and H-G) independently reviewed the titles and abstracts to screen the potentially eligible articles. After examining the full text, the included studies were finally confirmed. The following information was extracted by two independent investigators (XM-W and H-G): the authors, country, year of publication, number of included RCTs and participants contained, intervention, comparison, risk of bias assessment tool, outcomes, overall conclusions, and relevant data of the included RCTs. Any discrepancies in these two workflows were settled by consultation and arbitration with the third investigator (LL-R).

### Assessment of methodological quality

The methodological quality of the included SRs/MAs was evaluated by independent investigators (RM-L and XM-W) utilizing A Measure Tool to Assess Systematic Reviews 2 (AMSTAR-2) (34). The critical areas were assessed by seven items (2, 4, 7, 9, 11, 13, 15). Each item was classified as “no,” “partial yes,” or “yes” based on its conformance to the criteria. The overall level of methodological quality was categorized as “high,” “moderate,” “low,” or “critically low.” Disparities that arose throughout the evaluation were resolved through discussion by a third investigator (LL-R).

### Assessment of risk of bias

In this umbrella review, the risk of bias in the review process, the results, and the conclusions of included SRs/MAs was determined by investigators (RM-L and HW-Q) with the assistance of The Risk of Bias in Systematic Review (ROBIS) scale (35, 36). The scale was completed in three phases: (a) assessing relevance, (b) identifying concerns with the review process, and (c) judging the risk of bias. Throughout the evaluation, discrepancies were resolved through discussion by a third investigator (LL-R).

### Assessment of reporting quality

The reporting quality was evaluated by independent investigators (HW-Q and XM-W) using the 27-item Preferred Reporting Items for Systematic Reviews and Meta-Analyses (PRISMA) (37). There were two possible responses for each item: “yes” or “no”. Points were given based on each response. Any disagreements that arose over the process of the assessment were discussed and settled by a third investigator (WL-G).

### Assessment of quality of evidence

The evidence quality for outcomes were assessed by The Grading of Recommendations Assessment, Development, and Evaluation (GRADE) system (38). Bias risk, inconsistency, indirectness, imprecision, and publication bias were five factors that could lower the quality of the evidence. There were four levels of evidence quality in this system: high, moderate, low, and very low. XM-W and HW-Q were responsible for the specific evaluation, whereas WL-G was responsible for discussing and settling any disagreements in this process.

### Statistical analysis

A considerable number of SRs/MAs published in a short time frame that concentrate on the same field may contain numerous duplicate RCTs, which may introduce bias into the overall results. To evaluate the possible effect caused by including the same RCTs, we calculated the amount of overlap using the corrected covered area (CCA). The primary RCTs served as rows and the included SRs/MAs served as columns in the matrix, as described by Pieper et al. (39). The total number of RCTs included in SRs/MAs, RCTs, and included SRs/Mas were denoted by “*N*” (repetition permitted), “*r*,” and “*c*,” then CCA = (*N* − *r*)/[(*r* × *c*) − *r*]. Minor overlap was indicated by a CCA value between 0% and 5%, moderate overlap by 6% to 10%, high overlap by 11% to 15%, and very high overlap by >15% (40).

To determine if the significance of combined effect size was due to chance or bias, the excess significance tests on categorical variable outcomes in the included SRs/MAs were performed. Excess significance bias was determined by comparing the observed number (O) with the expected number (E); the greater the discrepancy between the two values, the more severe the bias. A *P*-value of less than 0.10 suggested an excess significance for a single SR/MA (32, 41).

Utilizing data from individual RCTs, we repooled various outcome indicators with incongruent SR/MA effect sizes [e.g., risk ratio (RR), hazard ratio (HR), odds ratio (OR), or standard mean difference (SMD); when applicable, the confidence interval (CI) was also calculated]. At the same time, for participants with type 2 diabetes (T2D), chronic kidney disease (CKD), acute heart failure (AHF), non-AHF, and intervention with SGLT-1/2is or SGLT-2is, we conducted subgroup meta-analyses to determine the potential sources of heterogeneity. The significance threshold was established at *P* < 0.05. In cases where no heterogeneity was identified, a fixed effects model was utilized, whereas the random effects model was used otherwise. If the *P*-value for the *Q* test was less than 0.10 and the *I*^2^ value was more than 25%, then we concluded that there existed heterogeneity (42). Egger's test was utilized to assess the evidence for small-study effects (43). Additionally, we performed a sensitivity analysis to assess the robustness and reliability of the combined results. R 4.1.1 (http://www.R-project.org) and Stata 16.0 (StataCorp LLC) were utilized for all statistical analyses.

## Results

### Search results

**Figure 1:**
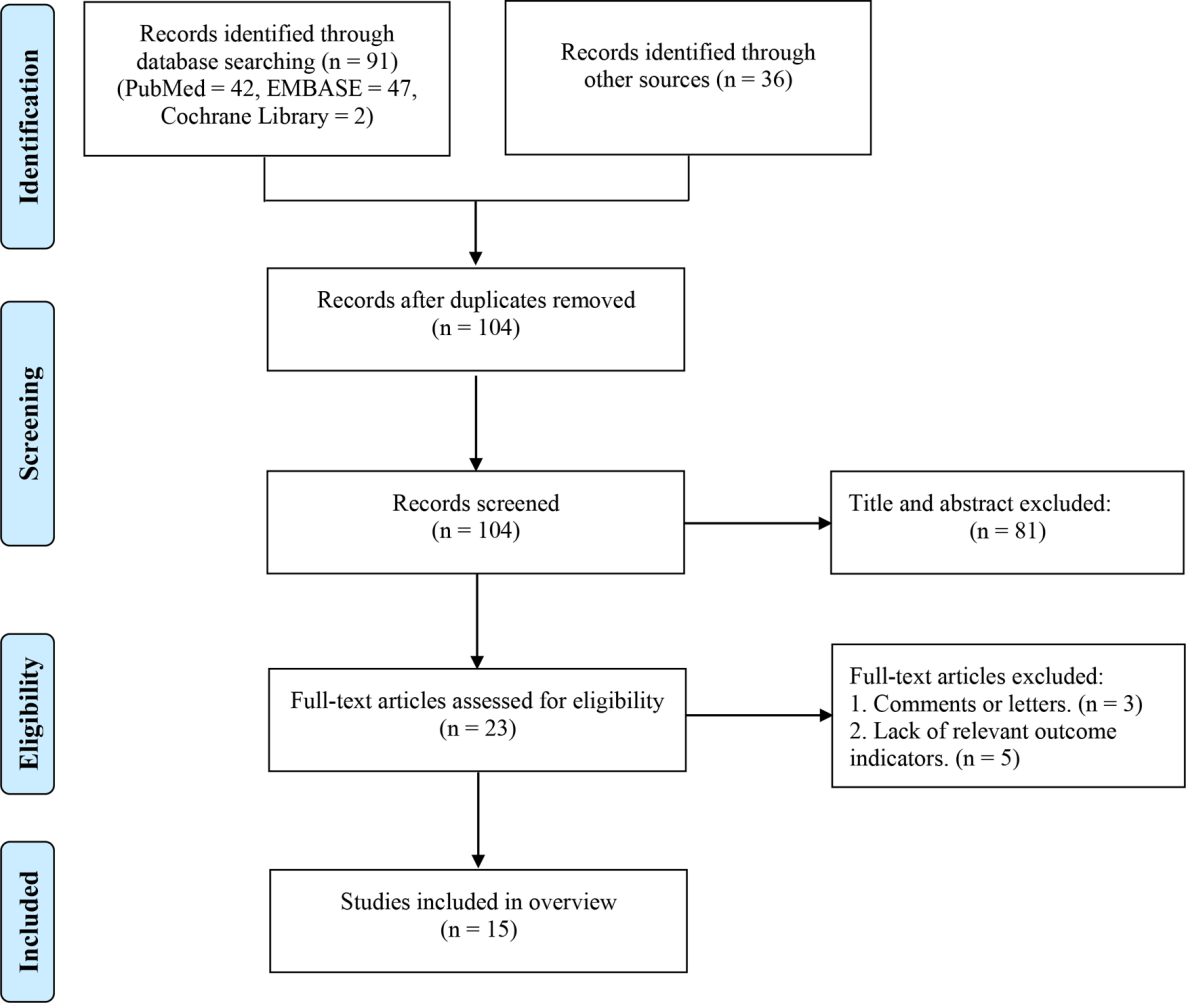
Flowchart of the screening process.

Following the research strategy, 127 potentially relevant records were obtained, and 23 of them were excluded after eliminating duplicates. After title and abstract-based screening, we eliminated 81 records. The remaining 23 records were then retrieved for full-text evaluation. Eight records were eliminated at this stage. Finally, 15 records (44–58) were included in this umbrella review. Figure 1 showed the literature screening procedure. Supplementary Table S2 provided detailed information on the excluded literature.

### Characteristics of included SRs/MAs

The characteristics of the 15 SRs/MAs were summarized in Table 1. A total of 17 primary RCTs (20–28, 59–70) were included across 15 SRs/MAs, and their corresponding relationships were shown in Supplementary Table S3. The overall CCA value was 23.53%, indicating a very high level of overlap. This suggested that a considerable amount of attention was devoted to research on SGLT-2i treatment of HFpEF, and there was a lack of relevant RCTs. The specific calculation process was also shown in Supplementary Table S3. The included SRs/MAs had a range of 2 to 12 RCTs, with sample sizes from 1,810 to 15,989 participants per trial, and all were published between 2020 and 2022. All included SRs/MAs were published in English, and the researchers were mainly from Asia and North America. Seven SRs/MAs were performed in China (45, 46, 42–54, 57, 58), 4 in The United States (44, 48, 50, 51), and one each in India (47), Canada (49), Japan (55), and United Kingdom (57). Regarding intervention modality, the control group was given CT or placebo, whereas the intervention group was given various types of SGLT-2is, including “Canagliflozin,” “Dapagliflozin,” “Empagliflozin,” “Ertugliflozin,” “Ipragliflozin,” “Luseogliflozin,” and “Sotagliflozin”. For the risk and bias assessment of the included RCTs, all the SRs/MAs selected the Cochrane criteria except Jhund et al. (57).

**Table 1:** Characteristics of the included SRs/MAs.

Author, year (Country)	Trials (participants)	Intervention group	Control group	Risk of bias assessment tool	Main results
Butler J, 2020 (USA) (44)	3 (2,554)	Dapagliflozin Ertugliflozin Sotagliflozin	Placebo	Cochrane Criteria	A significant reduction in first HHF and the composite of HHF or CVD was observed with SGLT-2is use. Upon sensitivity analysis by excluding SOLOIST-WHF/SCORED data, the results became non-significant but continued to exhibit a trend towards a benefit with SGLT-2is. However, there was no significant difference between the SGLT-2is and placebo groups in the incidence of CVD and all-cause death among HFpEF patients was noted between the SGLT-2is and control groups.
Lu Y, 2021 (CHN) (45)	2 (1,810)	Dapagliflozin Sotagliflozin	Placebo	Cochrane Criteria	The use of SGLT-2is only had a strong trend to be associated with a lower risk of CVD or HHF compared with placebo in HFpEF patients.
Zheng CY, 2021 (CHN) (46)	2 (2,323)	Dapagliflozin Ertugliflozin	Placebo	Cochrane Criteria	There were no significant differences in all-cause death, CVD, or total HHF were noted between the SGLT-2is and control groups among patients with HFpEF.
Singh A, 2021 (IND) (47)	4 (3,738)	Dapagliflozin Ertugliflozin Sotagliflozin	Placebo	Cochrane Criteria	SGLT-2is reduced the risk of the composite of HHF or CVD.
Cardoso R, 2021 (USA) (48)	4 (3,738)	Dapagliflozin Ertugliflozin Sotagliflozin	Placebo	Cochrane Criteria	In the subgroup of HFpEF, there was a 25% relative risk reduction in the composite of HHF or CVD among those treated with SGLT-2is compared with placebo.
Pandey A, 2022 (CNA) (49)	2 (6,482)	Empagliflozin Sotagliflozin	Placebo	Cochrane Criteria	In the subgroup of patients with HFpEF, significant reductions in the composite outcome of HHF and CVD were observed.
Vaduganathan M, 2022 (USA) (50)	2 (12,251)	Dapagliflozin Empagliflozin	Placebo	Cochrane Criteria	Compared with placebo, SGLT-2is in patients with HFpEF were associated with a statistically significant lower risk of the composite of HHF or CVD and first HHF. In the intervention group, there was greater improvement of the KCCQ-TSS, KCCQ-CSS, and KCCQ-OSS. However, there were no significant differences in terms of CVD and all-cause death was observed. Studies of adverse events showed that the intervention group had fewer cases of amputation, diabetic ketoacidosis, hypoglycemia, glycemia, renal events, and any serious adverse events compared with the control group. Meta-analyses were not performed due to differences in the definition of adverse events among RCTs.
Razuk V, 2022 (USA) (51)	4 (5,936)	Empagliflozin Sotagliflozin	Placebo	Cochrane Criteria	Among patients with HFpEF, SGLT-2is were associated with a reduced risk of the composite of HHF or CVD.
Cao Y, 2022 (CHN) (52)	5 (10,892)	Dapagliflozin Ertugliflozin Empagliflozin Sotagliflozin	Placebo	Cochrane Criteria	The effect of SGLT-2is in HFpEF patients tended to be beneficial in terms of the composite of HHF or CVD, total HHF or CVD, and first HHF. However, there were no statistically significant differences in CVD or any death were noted.
Zhao LY, 2022 (CHN) (53)	6 (10,550)	Dapagliflozin Ertugliflozin Empagliflozin Sotagliflozin	Conventional treatment or Placebo	Cochrane Criteria	The results showed that the incidence of first HHF and the composite outcome of HHF or CVD were lower in the SGLT-2is group compared with the control group among patients with HFpEF. However, no difference in the CVD rate was noted between the two groups.
Yang DN, 2022 (CHN) (54)	10 (10,334)	Canagliflozin Dapagliflozin Ertugliflozin Empagliflozin Sotagliflozin	Conventional treatment or Placebo	Cochrane Criteria	A meta-analysis revealed that SGLT-2is treatment reduced the incidence of the composite outcome (CVD or HHF) and total HHF, but there were no advantages in reducing CVD and all-cause death. The SGLT-2is group had a larger decrease in KCCQ-TSS from baseline than the placebo group, but no differences in KCCQ-PL, KCCQ-CSS, KCCQ-OSS, 6MWD, and NT-proBNP were observed.
Fukuta H, 2022 (JPN) (55)	11 (10,845)	Canagliflozin Dapagliflozin Ertugliflozin Empagliflozin Ipragliflozin Luseogliflozin Sotagliflozin	Conventional treatment or Placebo	Cochrane Criteria	SGLT-2is reduced the risk of a composite of HHF and CVD and the risk of total HHF. SGLT-2is did not reduce the risk of CVD or the risk of all-cause death. SGLT-2is decreased NT-proBNP levels and increased 6MWD, hematocrit levels, and KCCQ-TSS compared with the control group. However, SGLT-2is did not change BNP levels compared with the control group.
Zhou HF, 2022 (CHN) (56)	12 (10,883)	Canagliflozin Dapagliflozin Ertugliflozin Empagliflozin Luseogliflozin Sotagliflozin	Conventional treatment or Placebo	Cochrane Criteria	SGLT-2is significantly reduced the adverse events, the number of first HHF, the total HHF, the *E*/*e*′, and the composite of first HHF or CVD compared to placebo in patients with HFpEF. However, no differences in CVD, all-cause death, NT-proBNP, BNP, or 6MWD were noted between the two groups. The incidence of adverse events was significantly lower in the intervention group compared with the control group.
Jhund PS, 2022 (UK) (57)	2 (6,263)	Dapagliflozin	Placebo	—	In patients with HF, irrespective of left ventricular ejection fraction, Dapagliflozin led to significant reductions in the risk of CVD and all-cause death.
Wang YT, 2022 (CHN) (58)	6 (15,989)	Dapagliflozin Ertugliflozin Empagliflozin Sotagliflozin	Placebo	Cochrane Criteria	This meta-analysis of patients with HFpEF showed that SGLT-2i significantly reduced the risk of the composite of CVD and HHF, but not CVD and all-cause death. The incidence of adverse events was significantly lower in the intervention group compared with the control group.

SRs/MAs, systematic reviews and meta-analyses; SGLT-2i, sodium-glucose cotransporter-2 inhibitor; HFpEF, heart failure with preserved ejection fraction; HHF, hospitalization for heart failure; CVD, cardiovascular death; RCTs, randomized controlled trials; BNP, B-type natriuretic peptide; NT-proBNP, N-terminal pro-B-type natriuretic peptide; 6MWD, 6 min-walk distance; KCCQ-TSS, the Kansas City Cardiomyopathy Questionnaire Total Symptom Score; KCCQ-CSS, the Kansas City Cardiomyopathy Questionnaire Clinical Summary Score; KCCQ-OSS, the Kansas City Cardiomyopathy Questionnaire Overall Summary Score; KCCQ-PL, the Kansas City Cardiomyopathy Questionnaire Physical Limitation; *E*/*e*′, the ratio of early mitral inflow velocity to mitral annular early diastolic velocity. CHN, China; CAN, Canada; IND, India; JPN, Japan; UK, United Kingdom; USA, United States of America.

### Methodological quality assessment

Seven SRs/MAs were assessed as critically low quality (44, 49, 50, 54, 55, 57, 58), 7 were assessed as low quality (45–48, 51–53), and 1 was assessed as high quality (56) using the AMSTAR-2. Item 2 [lack of protocol before the study (10/15, 66.67%)], Item 7 [lack of excluded trials list (14/15, 93.33%)], and Item 15 [lack of an adequate investigation and discussion of publication bias (7/15, 46.67%)] were the most common absence of the 7 critical items. Table 2 provided the evaluation results of the AMSTAR-2 assessment for each study.

**Table 2:** Results of the AMSTAR-2 assessments.

Author, year (Country)	Q1	Q2	Q3	Q4	Q5	Q6	Q7	Q8	Q9	Q10	Q11	Q12	Q13	Q14	Q15	Q16	Quality
Butler J, 2020 (USA) (44)	Y	PY	Y	Y	Y	Y	N	Y	Y	Y	Y	Y	Y	N	N	Y	CL
Lu Y, 2021 (CHN) (45)	Y	PY	Y	Y	Y	Y	N	Y	Y	Y	Y	Y	Y	Y	Y	Y	L
Zheng CY, 2021 (CHN) (46)	Y	PY	Y	Y	Y	Y	N	Y	Y	Y	Y	Y	Y	Y	Y	Y	L
Singh A, 2021 (IND) (47)	Y	PY	Y	PY	Y	Y	N	Y	Y	Y	Y	Y	Y	Y	Y	Y	L
Cardoso R, 2021 (USA) (48)	Y	PY	Y	Y	Y	Y	N	Y	Y	Y	Y	Y	Y	Y	Y	Y	L
Pandey A, 2022 (CNA) (49)	Y	PY	Y	Y	Y	Y	N	Y	Y	Y	Y	Y	Y	N	N	Y	CL
Vaduganathan M, 2022 (USA) (50)	Y	Y	Y	Y	Y	Y	N	Y	Y	Y	Y	Y	Y	N	N	Y	CL
Razuk V, 2022 (USA) (51)	Y	Y	Y	PY	Y	Y	N	Y	Y	Y	Y	Y	Y	Y	Y	Y	L
Cao Y, 2022 (CHN) (52)	Y	PY	Y	Y	Y	Y	N	Y	Y	Y	Y	Y	Y	Y	Y	Y	L
Zhao LY, 2022 (CHN) (53)	Y	PY	Y	Y	Y	Y	N	Y	Y	Y	Y	Y	Y	Y	Y	Y	L
Yang DN, 2022 (CHN) (54)	Y	PY	Y	Y	Y	Y	N	Y	Y	Y	Y	Y	Y	N	N	Y	CL
Fukuta H, 2022 (JPN) (55)	Y	Y	Y	Y	Y	Y	N	Y	Y	Y	Y	Y	Y	N	N	Y	CL
Zhou HF, 2022 (CHN) (56)	Y	Y	Y	Y	Y	Y	Y	Y	Y	Y	Y	Y	Y	Y	Y	Y	H
Jhund PS, 2022 (UK) (57)	Y	PY	Y	PY	N	N	N	Y	N	Y	Y	Y	Y	N	N	Y	CL
Wang YT, 2022 (CHN) (58)	Y	Y	Y	PY	Y	Y	N	Y	Y	Y	Y	Y	Y	Y	N	Y	CL

Y, yes; PY, partial yes; N, no; CL, critically low; L, low; H, high. CHN, China; CAN, Canada; IND, India; JPN, Japan; UK, United Kingdom; USA, United States of America.

Q1: Did the research questions and inclusion criteria for the review include the components of PICO?

Q2: Did the report of the review contain an explicit statement that the review methods were established prior to the conduct of the review and did the report justify any significant deviations from the protocol?

Q3: Did the review authors explain their selection of the study designs for inclusion in the review?

Q4: Did the review authors use a comprehensive literature search strategy?

Q5: Did the review authors perform study selection in duplicate?

Q6: Did the review authors perform data extraction in duplicate?

Q7: Did the review authors provide a list of excluded studies and justify the exclusions?

Q8: Did the review authors describe the included studies in adequate detail?

Q9: Did the review authors use a satisfactory technique for assessing the risk of bias (RoB) in individual studies that were included in the review?

Q10: Did the review authors report on the sources of funding for the studies included in the review?

Q11: If meta-analysis was performed did the review authors use appropriate methods for statistical combination of results?

Q12: If meta-analysis was performed, did the review authors assess the potential impact of RoB in individual studies on the results of the meta-analysis or other evidence synthesis?

Q13: Did the review authors account for RoB in individual studies when interpreting/discussing the results of the review?

Q14: Did the review authors provide a satisfactory explanation for, and discussion of, any heterogeneity observed in the results of the review?

Q15: If they performed quantitative synthesis did the review authors carry out an adequate investigation of publication bias (small study bias) and discuss its likely impact on the results of the review?

Q16: Did the review authors report any potential sources of conflict of interest, including any funding they received for conducting the review?

### Risk of bias assessment

Regarding the ROBIS evaluation outcomes, phase 1 examined the relevance of study topics, while phase 2 domain 1 evaluated study eligibility criteria. For both items, all SRs/MAs were assessed as low risk of bias. For the included SRs/MAs, in domain 2, 12 were assessed as low risk of bias (12/15, 80.00%) (44–46, 48–50, 52–56, 58), in domain 3, 11 were assessed as low risk of bias (11/15, 73.33%) (44, 46–51, 53, 55, 56, 58), and in domain 4, only 1 was assessed as low risk of bias (1/15, 6.67%) (56). In phase 3, 10 SRs/Mas had a low risk of bias (10/15, 66.67%) (45–48, 51–53, 56–58). The details of the ROBIS assessments were shown in Supplementary Table S4.

### Reporting quality assessment

Supplementary Table S5 provided details on the report quality. Despite the fact that the titles, introductions, and discussions of the SRs/MAs included in this umbrella review were reported completely, reporting problems were discovered in other aspects. In the abstract section, Item 12 (registration) had a 33.33% response rate. In the methods section, the response rates for Item 7 (search strategy), Item 13(e) and (f) (synthesis methods), Item 14 (reporting bias assessment), and Item 15 (certainty assessment) were 80.00%, 66.67%, 80.00%, 53.33%, and 20.00%, respectively. In the results section, Item 16(b) (study selection), Item 20(c) and (d) (results of syntheses), Item 21 (reporting biases), and Item 22 (certainty of the evidence) exhibited less than 80% response rates. The quality assessment of Items 24(a) and (b) (registration and protocol) was inadequate since only 5 (50, 51, 55, 56, 58) (5/15, 33.33%) SRs/MAs provided information on research protocol registration.

### Evidence quality assessment

Table 3 summarized the results of the evidence quality assessment for 70 outcomes among the 15 included SRs/MAs. The evidence quality was assessed as very low in 11 cases (11/70, 15.71%), low in 36 cases (36/70, 51.43%), moderate in 22 cases (22/70, 31.43%), and high in one case (1/70, 1.43%). Publication bias (*n* = 69) was the most prevalent factor for downgrading, followed by imprecision (*n* = 47) and inconsistency (*n* = 11). Table 3 provided details on downgrades for each GRADE domain by the outcome.

**Table 3:** Results of evidence quality assessments.

Citation	Outcomes	Limitations	Inconsistency	Indirectness	Imprecision	Publication bias	Quality
Butler J, 2020 (USA) (44)	Composite of HHF or CVD	0	0	0	−1 ②	−1 ③	Low
	First HHF	0	0	0	−1 ②	−1 ③	Low
	CVD	0	0	0	−1 ②	−1 ③	Low
	All-cause death	0	0	0	−1 ②	−1 ③	Low
Lu Y, 2021 (CHN) (45)	Composite of HHF or CVD	0	0	0	−1 ②	−1 ③	Low
Zheng CY, 2021 (CHN) (46)	Total HHF	0	0	0	−1 ②	−1 ③	Low
	CVD	0	0	0	−1 ②	−1 ③	Low
	All-cause death	0	0	0	−1 ②	−1 ③	Low
Singh A, 2021 (IND) (47)	Composite of HHF or CVD	0	0	0	−1 ②	−1 ③	Low
Cardoso R, 2021 (USA) (48)	Composite of HHF or CVD	0	0	0	−1 ②	−1 ③	Low
Pandey A, 2022 (CNA) (49)	Composite of HHF or CVD	0	0	0	−1 ②	−1 ③	Low
Vaduganathan M, 2022 (USA) (50)	Composite of HHF or CVD	0	0	0	0	−1 ③	Moderate
	CVD	0	0	0	0	−1 ③	Moderate
	First HHF	0	0	0	0	−1 ③	Moderate
	All-cause death	0	0	0	0	−1 ③	Moderate
	≥5 points improvement in KCCQ-TSS	0	0	0	0	−1 ③	Moderate
	≥5 points improvement in KCCQ-CSS	0	0	0	0	−1 ③	Moderate
	≥5 points improvement in KCCQ-OSS	0	0	0	0	−1 ③	Moderate
	≥5 points deterioration in KCCQ-TSS	0	0	0	0	−1 ③	Moderate
	≥5 points deterioration in KCCQ-CSS	0	0	0	0	−1 ③	Moderate
	≥5 points deterioration in KCCQ-OSS	0	0	0	0	−1 ③	Moderate
Razuk V, 2022 (USA) (51)	Composite of HHF or CVD	0	0	0	−1 ②	−1 ③	Low
Cao Y, 2022 (CHN) (52)	Composite of first HHF or CVD	0	0	0	−1 ②	−1 ③	Low
	First HHF	0	0	0	−1 ②	−1 ③	Low
	CVD	0	0	0	−1 ②	−1 ③	Low
	All-cause death	0	0	0	−1 ②	−1 ③	Low
	Total HHF or CVD	0	0	0	0	−1 ③	Moderate
Zhao LY, 2022 (CHN) (53)	CVD	0	0	0	−1 ②	−1 ③	Low
	Composite of HHF or CVD	0	0	0	−1 ②	−1 ③	Low
	First HHF	0	0	0	−1 ②	−1 ③	Low
Yang DN, 2022 (CHN) (54)	Composite of HHF and CVD	0	0	0	0	−1 ③	Moderate
	CVD	0	0	0	−1 ②	−1 ③	Low
	Total HHF	0	0	0	0	−1 ③	Moderate
	All-cause death	0	0	0	−1 ②	−1 ③	Low
	KCCQ-TSS	0	0	0	0	−1 ③	Moderate
	KCCQ-PL	0	−1 ①	0	−1 ②	−1 ③	Very low
	KCCQ-CSS	0	−1 ①	0	−1 ②	−1 ③	Very low
	KCCQ-OSS	0	−1 ①	0	−1 ②	−1 ③	Very low
	6MWD	0	−1 ①	0	−1 ②	−1 ③	Very low
	NT-ProBNP	0	−1 ①	0	−1 ②	−1 ③	Very low
Fukuta H, 2022 (JPN) (55)	Composite of HHF and CVD	0	0	0	0	−1 ③	Moderate
	Total HHF	0	0	0	−1 ②	−1 ③	Low
	CVD	0	0	0	−1 ②	−1 ③	Low
	All-cause death	0	0	0	−1 ②	−1 ③	Low
	NT-ProBNP	0	0	0	−1 ②	−1 ③	Low
	BNP	0	0	0	−1 ②	−1 ③	Low
	6MWD	0	0	0	0	−1 ③	Moderate
	KCCQ-TSS	0	−1 ①	0	−1 ②	−1 ③	Very low
	Hematocrit	0	0	0	−1 ②	−1 ③	Low
Zhou HF, 2022 (CHN) (56)	Composite of HHF or CVD	0	0	0	0	0	High
	First HHF	0	0	0	0	−1 ③	Moderate
	CVD	0	0	0	−1 ②	−1 ③	Low
	Total HHF	0	0	0	−1 ②	−1 ③	Low
	All-cause death	0	0	0	−1 ②	−1 ③	Low
	*E*/*e*′	0	−1 ①	0	−1 ②	−1 ③	Very low
	The change of NT-proBNP	0	−1 ①	0	−1 ②	−1 ③	Very low
	NT-proBNP	0	0	0	−1 ②	−1 ③	Low
	BNP	0	−1 ①	0	−1 ②	−1 ③	Very low
	6MWD	0	−1 ①	0	−1 ②	−1 ③	Very low
	Adverse events	0	0	0	−1 ②	−1 ③	Low
Jhund PS, 2022 (UK) (57)	CVD	0	0	0	−1 ②	−1 ③	Low
	All-cause death	0	0	0	−1 ②	−1 ③	Low
	Total HHF	0	0	0	0	−1 ③	Moderate
	First HHF	0	0	0	−1 ②	−1 ③	Low
	Composite of HHF or CVD	0	0	0	0	−1 ③	Moderate
Wang YT, 2022 (CHN) (58)	Composite of HHF or CVD	0	0	0	0	−1 ③	Moderate
	CVD	0	−1 ①	0	−1 ②	−1 ③	Very low
	Total HHF	0	0	0	0	−1 ③	Moderate
	All-cause death	0	0	0	−1 ②	−1 ③	Low
	Adverse events	0	0	0	0	−1 ③	Moderate

① The confidence interval overlaps less or the *I*^2^ value of the combined results was larger. ② The sample size from the included studies did not meet the optimal sample size or the 95% confidence interval crosses the invalid line. ③ Funnel plots were missing or asymmetrical. HHF, hospitalization for heart failure; CVD, cardiovascular death; BNP, B-type natriuretic peptide; NT-proBNP, N-terminal pro-B-type natriuretic peptide; 6MWD, 6 min-walk distance; KCCQ-TSS, the Kansas City Cardiomyopathy Questionnaire Total Symptom Score; KCCQ-CSS, the Kansas City Cardiomyopathy Questionnaire Clinical Summary Score; KCCQ-OSS, the Kansas City Cardiomyopathy Questionnaire Overall Summary Score; KCCQ-PL, the Kansas City Cardiomyopathy Questionnaire Physical Limitation; *E*/*e*′, the ratio of early mitral inflow velocity to mitral annular early diastolic velocity. CHN, China; CAN, Canada; IND, India; JPN, Japan; UK, United Kingdom; USA, United States of America.

### Summary and reestimation of outcome indicators

**Figure 2:**
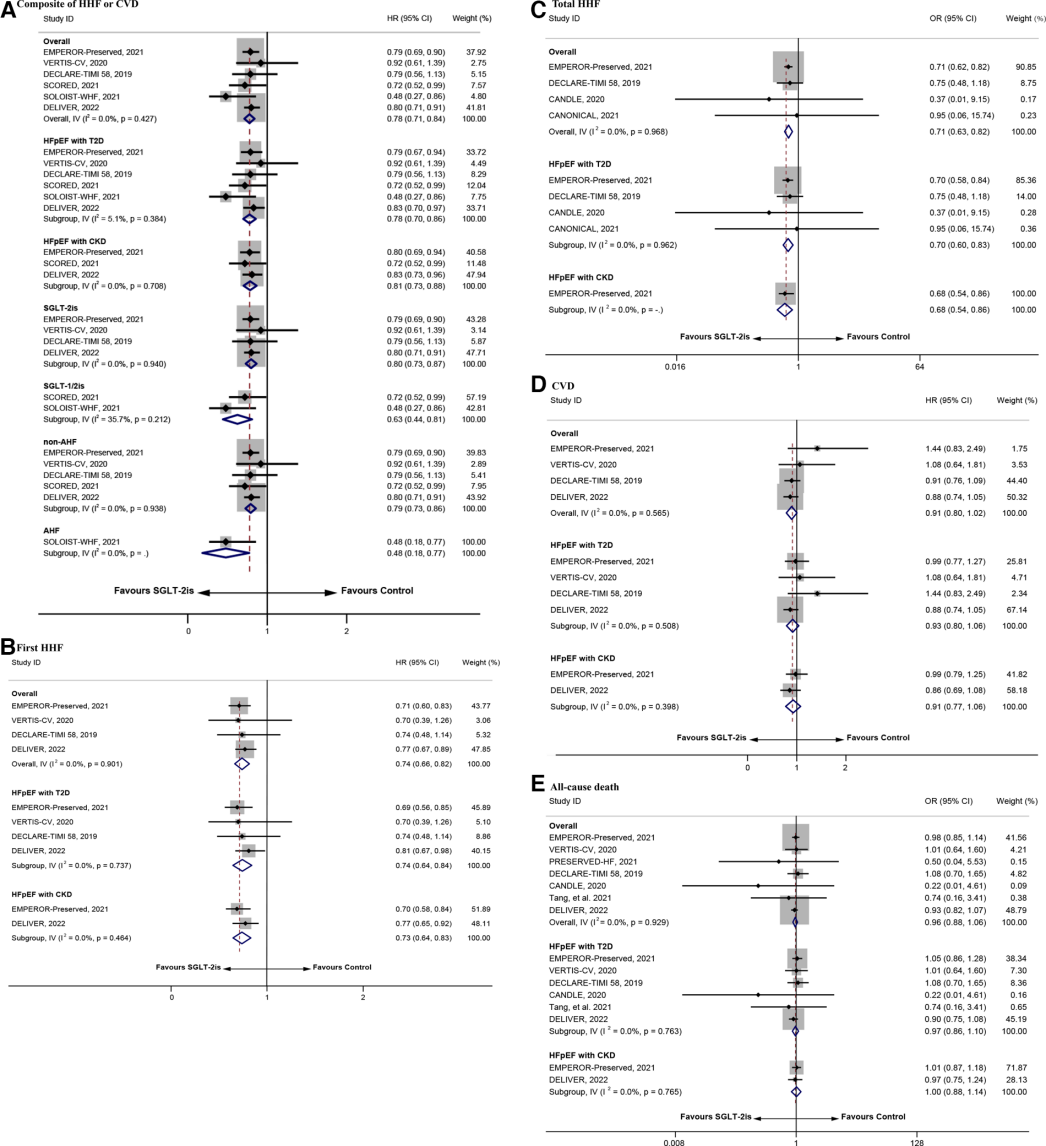
The repooled effect size of the composite of HHF or CVD, first HHF, total HHF, CVD, and all-cause death. HHF, hospitalization for heart failure; CVD, cardiovascular death.

**Figure 3:**
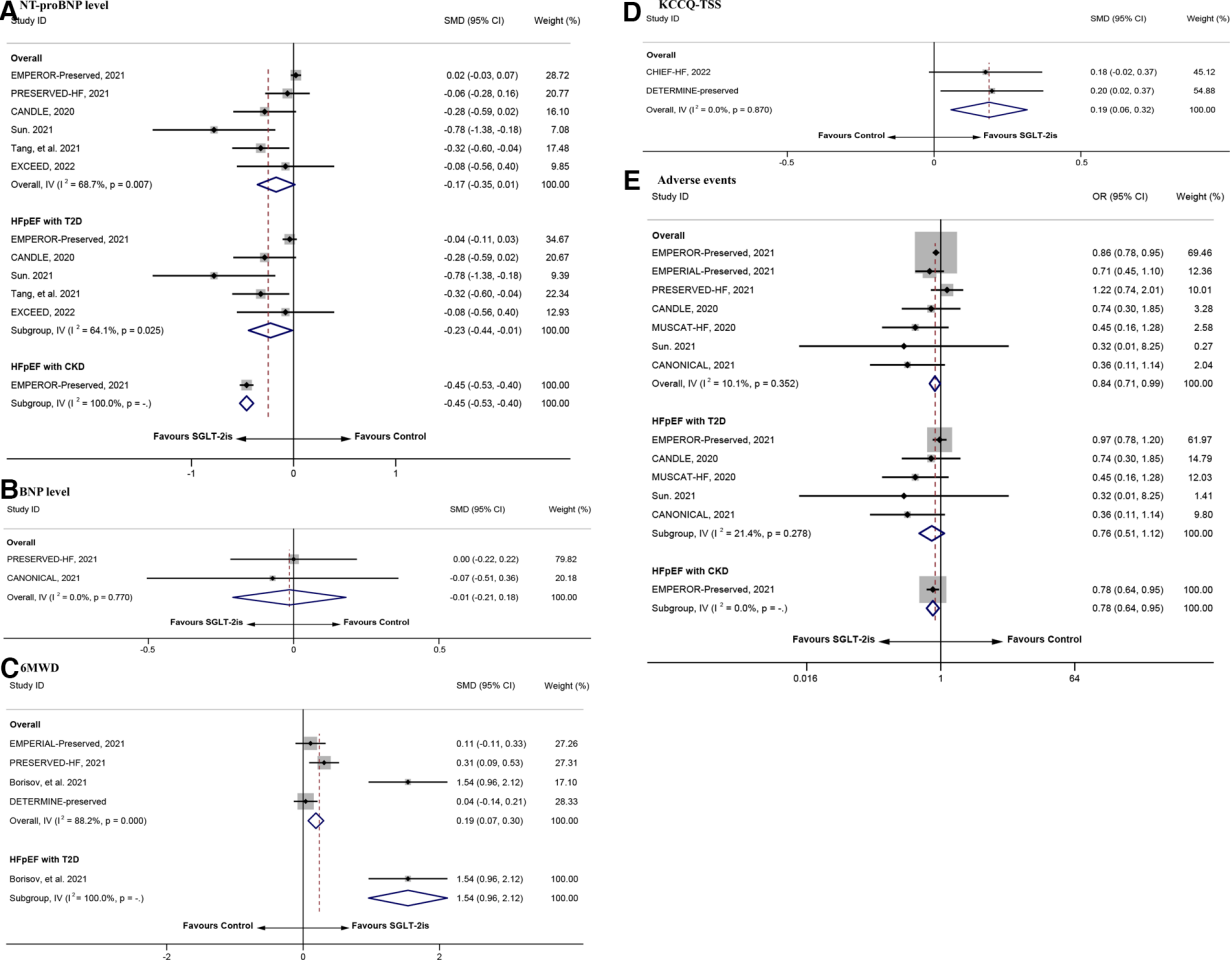
The repooled effect size of NT-proBNP level, BNP level, KCCQ-TSS, 6MWD, and adverse events. BNP, B-type natriuretic peptide; NT-proBNP, N-terminal pro-B-type natriuretic peptide; 6MWD: 6 min-walk distance; KCCQ-TSS: the Kansas City Cardiomyopathy Questionnaire Total Symptom Score.

In this umbrella review, we conducted a test for excess significance effect, a narrative description, and a reestimation of the quantitatively assessed outcome indicators by the SRs/MAs. Detailed information was provided in Table 4 and Figures 2, 3.

**Table 4:** Summary of evidence and excess significance tests.

Citation	Outcomes	*I* ^2^	*P* for heterogeneity	Relative effect (95% CI)	*P* value	Excess significance tests		
						*O*/*E*	*χ* ^2^	*P*
Butler J, 2020 (USA) (44)	Composite of HHF or CVD	29.00%	0.25	HR = 0.80 (0.63–1.00)	0.05	0/0.45	0.81	0.82
	First HHF	0.00%	0.94	HR = 0.71 (0.52–0.97)	0.03	1/0.58	0.73	0.20
	CVD	0.00%	0.43	HR = 1.27 (0.92–1.76)	0.15	0/0.31	0.44	0.75
	All-cause death	0.00%	0.97	HR = 1.02 (0.79–1.30)	0.90	0/0.05	0.06	0.59
Lu Y, 2021 (CHN) (45)	Composite of HHF or CVD	1.00%	0.32	HR = 0.81 (0.64–1.02)	0.08	0/0.42	0.72	0.80
Zheng CY, 2021 (CHN) (46)	Total HHF	0.00%	0.86	RR = 0.74 (0.55–1.00)	0.05	0/0.12	0.14	0.64
	CVD	0.00%	0.41	RR = 1.26 (0.93–1.73)	0.14	0/0.09	0.10	0.62
	All-cause death	0.00%	0.96	RR = 1.02 (0.81–1.29)	0.87	0/0.05	0.05	0.59
Singh A, 2021 (IND) (47)	Composite of HHF or CVD	11.00%	0.34	HR = 0.75 (0.62–0.91)	<0.01	1/0.84	0.19	0.33
Cardoso R, 2021 (USA) (48)	Composite of HHF or CVD	11.00%	0.34	HR = 0.75 (0.62–0.91)	<0.01	1/0.84	0.19	0.33
Pandey A, 2022 (CNA) (49)	Composite of HHF or CVD	0.00%	0.50	HR = 0.78 (0.68–0.89)	<0.01	1/0.94	1.06	0.40
Vaduganathan M, 2022 (USA) (50)	Composite of HHF or CVD	–	0.89	HR = 0.80 (0.73–0.87)	<0.01	1/0.99	0	0.48
	CVD	–	1.00	HR = 0.88 (0.77–1.00)	0.05	0/0.47	0.89	0.83
	First HHF	–	0.46	HR = 0.74 (0.67–0.83)	<0.01	1/1	0	0.50
	All-cause death	–	0.52	HR = 0.97 (0.88–1.06)	0.48	0/0.09	0.10	0.63
	≥5 points improvement in KCCQ-TSS	–	0.92	OR = 1.17 (1.08–1.26)	<0.01	0/0.05	0.05	0.59
	≥5 points improvement in KCCQ-CSS	–	0.67	OR = 1.15 (1.06–1.24)	<0.01	0/0.05	0.05	0.59
	≥5 points improvement in KCCQ-OSS	–	0.41	OR = 1.16 (1.07–1.26)	<0.01	0/0.05	0.05	0.59
	≥5 points deterioration in KCCQ-TSS	–	0.59	OR = 0.78 (0.72–0.86)	<0.01	0/0.05	0.05	0.59
	≥5 points deterioration in KCCQ-CSS	–	0.28	OR = 0.80 (0.73–0.87)	<0.01	0/0.05	0.05	0.59
	≥5 points deterioration in KCCQ-OSS	–	0.69	OR = 0.80 (0.73–0.87)	<0.01	0/0.05	0.05	0.59
Razuk V, 2022 (USA) (51)	Composite of HHF or CVD	0.00%	0.71	HR = 0.80 (0.70–0.92)	<0.01	1/0.91	0.10	0.38
Cao Y, 2022 (CHN) (52)	Composite of first HHF or CVD	0.00%	0.66	HR = 0.81 (0.73–0.91)	<0.01	1/0.98	0.02	0.44
	First HHF	0.00%	1.00	HR = 0.71 (0.62–0.82)	<0.01	1/0.99	0	0.48
	CVD	47.00%	0.15	HR = 0.99 (0.84–1.15)	0.86	0/0.05	0.05	0.59
	All-cause death	0.00%	0.99	HR = 0.99 (0.89–1.13)	0.95	0/0.05	0.06	0.59
	Total HHF or CVD	0.00%	0.73	HR = 0.61 (0.43–0.86)	<0.01	1/0.79	0.26	0.30
Zhao LY, 2022 (CHN) (53)	CVD	23.90%	0.27	HR = 1.01 (0.80–1.28)	0.94	0/0.05	0.05	0.59
	Composite of HHF or CVD	0.00%	0.47	HR = 0.78 (0.70–0.87)	<0.01	1/0.99	0	0.47
	First HHF	0.00%	0.55	HR = 0.74 (0.64–0.85)	<0.01	1/0.98	0.02	0.45
Yang DN, 2022 (CHN) (54)	Composite of HHF or CVD	31.60%	0.21	HR = 0.77 (0.65–0.91)	<0.01	1/0.86	0.17	0.34
	CVD	35.50%	0.21	OR = 1.02 (0.77–1.35)	0.89	0/0.05	0.05	0.59
	Total HHF	0.00%	0.97	OR = 0.71 (0.61–0.83)	<0.01	0/0.07	0.07	0.61
	All-cause death	0.00%	0.97	OR = 0.99 (0.87–1.13)	0.94	0/0.05	0.05	0.59
	KCCQ-TSS	30.90%	0.22	MD = 2.74 (1.30–4.18)	<0.01	*–*	*–*	*–*
	KCCQ-PL	64.30%	0.04	MD = 1.66 (−0.67 to 3.98)	0.16	*–*	*–*	*–*
	KCCQ-CSS	72.60%	0.03	MD = 2.74 (1.30–4.18)	0.13	*–*	*–*	*–*
	KCCQ-OSS	51.20%	0.13	MD = 1.66 (−0.29 to 3.62)	0.10	*–*	*–*	*–*
	6MWD	59.90%	0.08	MD = 6.70 (−2.31 to 15.71)	0.15	*–*	*–*	*–*
	NT-ProBNP	55.50%	0.11	SMD = 1.66 (−0.29 to 3.62)	0.39	*–*	*–*	*–*
Fukuta H, 2022 (JPN) (55)	Composite of HHF or CVD	0.00%	0.46	HR = 0.78 (0.70–0.87)	<0.01	1/0.99	0	0.47
	Total HHF	0.00%	0.99	OR = 0.71 (0.61–0.83)	<0.01	0/0.23	0.30	0.71
	CVD	36.00%	0.21	OR = 0.95 (0.80–1.13)	0.55	0/0.05	0.06	0.59
	All-cause death	0.00%	0.92	OR = 1.00 (0.87–1.13)	0.94	0/0.05	0.05	0.59
	NT-ProBNP	0.00%	0.89	WMD = −60.16 (−82.99 to 37.33)	<0.01	–	–	–
	BNP	0.00%	0.36	WMD = 7.53 (–22.87–7.82)	0.34	–	–	–
	6MWD	0.00%	0.66	WMD = 18.00 (6.80–29.30)	<0.01	–	–	–
	KCCQ-TSS	54.00%	0.11	WMD = 2.57 (0.19–4.96)	0.04	–	–	–
	Hematocrit	23.60%	0.25	WMD = 2.34 (2.16–2.51)	<0.01	–	–	–
Zhou HF, 2022 (CHN) (56)	Composite of HHF or CVD	0.00%	0.46	HR = 0.78 (0.70–0.87)	<0.01	1/0.99	0	0.47
	First HHF	0.00%	0.98	HR = 0.71 (0.62–0.83)	<0.01	1/0.99	0	0.48
	CVD	24.00%	0.27	HR = 0.96 (0.82–1.13)	0.63	0/0.08	0.08	0.62
	Total HHF	0.00%	0.97	HR = 0.75 (0.67–0.84)	<0.01	1/0.99	0	0.49
	All-cause death	0.00%	0.84	HR = 0.99 (0.88–1.11)	0.86	0/0.05	0.06	0.59
	*E*/*e*′	59.00%	0.12	MD = −1.22 (−2.29 to 0.15)	0.03	–	–	–
	The change of NT-proBNP	98.00%	–	MD = −26.60 (−61.20 to 7.99)	0.13	–	–	–
	NT-proBNP	0.00%	–	MD = −8.51 (−33.19 to 16.16)	0.50	–	–	–
	BNP	72.00%	–	MD = −21.04 (−75.69 to 33.62)	0.45	–	–	–
	6MWD	82.00%	–	MD = 14.99 (−4.60 to 34.60)	0.13	–	–	–
	Adverse events	10.00%	0.35	RR = 0.92 (0.88–0.97)	<0.01	0/0.08	0.09	0.62
Jhund PS, 2022 (UK) (57)	CVD (44%<LVEF ≤ 51%)	–	–	HR = 0.91 (0.69–1.20)	–	0/0.18	0.12	0.64
	CVD (51%<LVEF ≤ 60%)	–	–	HR = 1.02 (0.77–1.34)	–	0/0.06	0.06	0.60
	CVD (LVEF > 60%)	–	–	HR = 0.68 (0.47–1.00)	–	1/0.57	0.76	0.19
	All-cause death (44%<LVEF ≤ 51%)	–	–	HR = 0.94 (0.75–1.17)	–	0/0.10	0.11	0.63
	All-cause death (51%<LVEF ≤ 60%)	–	–	HR = 1.02 (0.82–1.27)	–	0/0.06	0.06	0.60
	All-cause death (LVEF > 60%)	–	–	HR = 0.86 (0.65–1.13)	–	0/0.25	0.33	0.72
	Total HHF (44%<LVEF ≤ 51%)	–	–	RR = 0.84 (0.68–1.03)	–	0/0.46	0.87	0.82
	Total HHF (51%<LVEF ≤ 60%)	–	–	RR = 0.63 (0.51–0.77)	–	0/0.45	0.88	0.80
	Total HHF (LVEF > 60%)	–	–	RR = 0.77 (0.59–1.02)	–	1/0.64	0.56	0.23
	First HHF (44%<LVEF ≤ 51%)	–	–	HR = 0.83 (0.64–1.07)	–	0/0.28	0.39	0.73
	First HHF (51%<LVEF ≤ 60%)	–	–	HR = 0.66 (0.51–0.84)	–	1/0.87	0.14	0.35
	First HHF (LVEF > 60%)	–	–	HR = 0.88 (0.64–1.22)	–	0/0.15	0.18	0.66
	Composite of HHF or CVD (44%<LVEF ≤ 51%)	–	–	HR = 0.90 (0.73–1.10)	–	0/0.14	0.17	0.64
	Composite of HHF or CVD (51%<LVEF ≤ 60%)	–	–	HR = 0.77 (0.63–0.95)	–	1/0.62	0.63	0.21
	Composite of HHF or CVD (LVEF > 60%)	–	–	HR = 0.77 (0.59–1.00)	–	1/0.60	0.65	0.20
Wang YT, 2022 (CHN) (58)	Composite of HHF or CVD	0.00%	0.59	HR = 0.79 (0.72–0.85)	<0.01	1/0.89	0.01	0.44
	CVD	6.00%	0.36	HR = 0.92 (0.82–1.04)	0.19	0/0.14	0.22	0.69
	Total HHF	0.00%	0.76	HR = 0.74 (0.67–0.82)	<0.01	1/0.94	0.01	0.49
	All-cause death	0.00%	0.89	HR = 0.97 (0.89–1.06)	0.55	0/0.16	0.20	0.67
	Adverse events	0.00%	0.35	HR = 0.89 (0.83–0.96)	<0.01	1/0.87	0.14	0.35

HHF, hospitalization for heart failure; CVD, cardiovascular death; BNP, B-type natriuretic peptide; NT-proBNP, N-terminal pro-B-type natriuretic peptide; 6MWD, 6 min-walk distance; KCCQ-TSS, the Kansas City Cardiomyopathy Questionnaire Total Symptom Score; KCCQ-CSS, the Kansas City Cardiomyopathy Questionnaire Clinical Summary Score; KCCQ-OSS, the Kansas City Cardiomyopathy Questionnaire Overall Summary Score; KCCQ-PL, the Kansas City Cardiomyopathy Questionnaire Physical Limitation; *E*/*e*′, the ratio of early mitral inflow velocity to mitral annular early diastolic velocity; LVEF, Left Ventricular Ejection Fractions; HR, hazard ratio; OR, odds ratio; RR, risk ratio; MD, mean difference; WMD, weighted mean difference; SMD, standardized mean difference; O, observed number; E, expected number. CHN, China; CAN, Canada; IND, India; JPN, Japan; UK, United Kingdom; USA, United States of America.

### Main outcome indicators

The main outcomes included first or total HHF, CVD, all-cause death, and the composite of HHF and CVD. No excess significant effects were found in the main outcome indicators (Table 4). There were 14 (44, 45, 47–58) SRs/MAs involving the composite of HHF or CVD. Eleven (47–56, 58) of these reviews indicated that SGLT-2is reduced the occurrence of this indicator relative to placebo or CT. Ten SRs/MAs (44, 46, 50, 52–58) reported the effect of SGLT-2is on CVD, but none of them found a significantly reduced CVD rate in patients with HFpEF. In addition to the MA from Jhund et al. (57), the remaining 5 SRs/MAs (44, 50, 52, 53, 56) demonstrated that SGLT-2is significantly reduce the incidence of first HHF. Four (54–57, 58) of the 6 (46, 54–58) SRs/MAs involving total HHF revealed that SGLT-2is were associated with a decrease in its incidence. In addition, none of the 9 SRs/MAs (44, 46, 50, 52, 54–58) supported the use of SGLT-2is to reduce all-cause death.

As shown in Figure 2, the repooled HRs (95% CI) were 0.78 (0.71–0.84) and 0.74 (0.66–0.82) for the composite of HHF or CVD and first HHF, respectively, suggesting that the risk in the intervention group was 22% and 26% lower than that in the control group. Subgroup analysis revealed that participants with diagnosis of T2D or CKD (HR = 0.78, 95% CI = 0.70–0.86 and HR = 0.81, 95% CI = 0.73–0.88), with diagnosis of non-AHF or AHF (HR = 0.79, 95% CI = 0.73–0.86 and HR = 0.48, 95% CI = 0.18–0.77), and treated with SGLT-2is or SGLT-1/2is (HR = 0.80, 95% CI = 0.73–0.87 and HR = 0.63, 95% CI = 0.44–0.81), had lower risk of the composite of HHF or CVD in the intervention group compared with the control group. Similar to the above results, participants with diagnosis of T2D or CKD (HR = 0.74, 95% CI = 0.64–0.84 and HR = 0.73, 95% CI = 0.64–0.83) had lower risk of first HHF in the intervention group. The repooled OR (95% CI) for total HHF was 0.71 (0.63–0.82), reflecting a 29% reduction in the total HHF rate with SGLT-2is. However, for CVD and all-cause death, there was no significant improvement in the intervention group (HR = 0.91, 95% CI = 0.80–1.02 and OR = 0.96, 95% CI = 0.88–1.06). Subgroup analysis revealed that participants with diagnosis of T2D or CKD in the intervention group had lower risk in the total HHF [OR = 0.70, 95% CI = 0.60–0.83 and OR = 0.68, 95% CI = 0.54–0.86)], but for participants with diagnosis of T2D or CKD, there were no significant improvement in CVD (HR = 0.93, 95% CI = 0.80–1.06 and HR = 0.91, 95% CI = 0.77–1.06) and all-cause death (OR = 0.97, 95% CI = 0.86–1.10 and OR = 1.00, 95% CI = 0.88–1.14) compared with the control group.

Egger's test showed no significant small-study effect on the composite of HHF or CVD, first HHF, total HHF, CVD, and all-cause death (Supplementary Table S6). The sensitivity analysis showed high reliability of the conclusions (Supplementary Figures S1A–E).

### Other outcome indicators

The markers of heart failure symptoms included NT-proBNP level, change in NT-proBNP, and BNP level. In the meanwhile, indicators of cardiac function outcomes included 6MWD and *E*/*e*′ level (Table 4). Two (54, 56) of the 3 SRs/MAs (54–56) that involved NT-proBNP levels found that SGLT-2is did not decrease the level of NT-proBNP. Furthermore, Zhou et al. (56) revealed that SGLT-2is were not associated with change in NT-proBNP. Similarly, 2 SRs/MAs (55, 56) reported that SGLT-2is did not significantly reduce BNP levels. The conclusions of the 3 SRs/MAs (54–56) on 6MWD were different. Fukuta et al. (55) suggested that SGLT-2is increased the 6MWD value, whereas Yang et al. (54) and Zhou et al. (56) reported the opposite results. In an extended study, Zhou et al. (56) found that SGLT-2is contribute to the reduction of *E*/*e*′ level.

Indicators of health status and life quality outcomes included the KCCQ-TSS, the KCCQ-PL, the KCCQ-CSS, and the KCCQ-OSS. All 3 SRs/MAs (50, 54, 55) showed that SGLT-2is increase the level of KCCQ-TSS, showing high consistency. Vaduganathan et al. (50) believed that SGLT-2is help improve the level of KCCQ-CSS and KCCQ-OSS, which was contrary to the research conclusion of Yang et al. (54). In addition, 1 review (54) showed no clear association between SGLT-2is and increased KCCQ-PL levels.

The research on adverse events by Vaduganathan et al. (50) showed that the intervention group had fewer cases of amputation, diabetic ketoacidosis, hypoglycemia, renal events, and any serious adverse events compared with the control group. MAs were not performed due to differences in the definition of adverse events among RCTs. Zhou et al. and Wang et al. (56, 58) demonstrated that the incidence of adverse events in the intervention group was significantly lower than that in the control group.

As shown in Figure 3, the repooled SMDs (95% CI) of NT-proBNP level, BNP level, 6MWD, and KCCQ-TSS were −0.17 (−0.35–0.01), −0.01 (−0.21–0.18), 0.19 (0.07–0.30), and 0.19 (0.06–0.32), respectively. Except for 6MWD and KCCQ-TSS, the above data did not support the significant positive effect of SGLT-2is on the outcome indicators (*P* > 0.05). Subgroup analysis revealed that participants with diagnosis of T2D or CKD in the intervention group had significant positive effect on NT-ProBNP level (SMD = −0.23, 95% CI = −0.44 to −0.01 and SMD = −0.45, 95% CI = −0.53 to −0.40). Similar to that, participants with diagnosis of T2D had significant positive effect on 6MWD (SMD = 1.54, 95% CI = 0.96–2.12). For adverse events, the incidence of adverse events was significantly lower in the intervention group than in the control group (OR = 0.84, 95% CI = 0.71–0.99). Similar conclusion was observed in the subgroup of patients with CKD (OR = 0.78, 95% CI = 0.64–0.95). However, this conclusion could not be applied to patients with T2D (OR = 0.76, 95% CI = 0.51–1.12). Moreover, Egger's test revealed no significant small-study effects for NT-proBNP level, 6MWD, and adverse events (Supplementary Table S6). The sensitivity analysis indicated that the conclusions were highly reliable (Supplementary Figure S1 F-J).

## Discussion

It is now increasingly recognized that the cardiovascular protective effects of SGLT-2is may be facilitated by blood pressure reductions, diuretic and natriuretic effects, attenuation of cardiac inflammation and fibrosis, reduction in left ventricular preload and afterload, improvement of endothelial function, reductions in oxidative stress and arterial stiffness (71, 72). In HFrEF, the effect of SGLT-2is on the composite outcome of HHF and CVD has been demonstrated. However, the efficacy of SGLT-2is in HFpEF remains controversial. For example, in terms of reducing the risk of first HHF and the composite of HHF or CVD, the outcomes of the EMPEROR-Preserved trial (20–22) are the opposite of the VERTIS-CV trial (23). In the meantime, SGLT-2is did not improve the value of 6WMD in the EMPERIAL-Preserved trial (24), which was opposite in the PRESERVED-HF trial (25). The EMPEROR-Preserved trial (20–22) found that SGLT-2is could reduce NT-ProBNP levels in patients with HFpEF, whereas the PRESERVED-HF trial (25) showed the opposite outcome. Numerous relevant SRs/MAs have been published to provide further evidence-based medical evidence, but their quality has not been evaluated. The current comprehensive umbrella review aimed to systematically assess the quality of SRs/MAs that examined the clinical efficacy of SGLT-2is in the treatment of HFpEF. We used the AMSTAR-2, ROBIS, PRISMA guidelines, GRADE framework, CCA, and excess significance tests to summarize and evaluate the dependability of study outcomes. Furthermore, to clarify the size and direction of the impact of SGLT-2is on patients with HFpEF, we repooled the primary RCTs of SRs/MAs to obtain updated conclusions. Our study provides methodological application warnings for relevant SR/MA studies and evidence-based medical evidence of the efficacy of SGLT-2is in treating HFpEF.

### Reasons for low evidence quality of current SRs/MAs

To the best of our knowledge, this study is the first umbrella review of SRs/MAs on the effects of SGLT-2is on HFpEF. The 15 included SRs/MAs were all published in the last three years, whereas the RCTs involved in each SR/MA were published in 2019 and later. This figure indicates that the therapeutic effect of SGLT-2is on HFpEF is becoming a research hotspot. However, as revealed by the assessment of methodological quality, risk of bias, reporting quality, and evidence quality, most of the included SRs/MAs were unsatisfactory.

The methodological quality assessment showed that 14 SRs/MAs performed poorly on the 7 critical items of the AMSTAR-2. These studies were classified into critically low quality or low quality, accounting for 93.33% of all the included SRs/MAs. The major shortcomings are highlighted as follows: (1) 10 SRs/MAs lacked research protocol registration, which may lead to significant modifications in the research process; weaken the standardization, rigor, and transparency of the SRs/MAs; and increase the possibility of selective report bias (73); (2) none of the 14 SRs/MAs offered an excluded literature list, reducing the transparency of the SRs/MAs and undermining the trustworthiness of the results; (3) although most studies evaluated publication bias, 7 SRs/MAs did not use funnel plots, Egger's test, Begg's test, and other tools to evaluate publication bias. This conclusion might be attributed to the lack of adequate RCTs for the outcome indicators, the irregular use of methodology still reduces the confidence in the findings; (4) additionally, regarding Item 14, 6 SRs/MAs did not analyze and discuss the heterogeneity of the RCT results and lacked necessary measures, such as subgroup analysis or meta-regression, which resulted in decreased reliability of the combined calculation. All of the mentioned methodological limitations reduce the reliability of SRs/MAs.

When reviewing the risk bias assessment results obtained by the ROBIS tool, it was found that the high-risk bias was mainly derived from Phase 2 domains 2–4 and Phase 3. For Phase 2, the high-risk bias mainly stems from the lack of an effective retrieval scheme for grey literature (domain 2); lack of partial research features (domain 3); lack of registration of the research protocol; lack of sensitivity analysis and heterogeneity intervention (domain 4); and inadequate assessment of publication bias (Phase 3). The above factors affect the authenticity and credibility of SR/MA results. Based on the PRISMA, the absence of protocol registration, publication bias, incomplete retrieval strategy, insufficient evaluation of heterogeneity, the lack of sensitivity analysis, and lack of certainty assessment of evidence for outcomes were important reasons to reduce the reporting quality of SRs/MAs.

The evidence quality assessment of 70 outcomes based on the GRADE tool showed that inconsistency, imprecision, and publication bias were the main factors of evidence grade reduction. Of these outcomes, the quality of evidence was very low for 11 (11/70, 15.71%), low for 36 (36/70, 51.43%), moderate for 22 (22/70, 31.43%), and high for 1 (1/70, 1.43%). Specifically, clinical and methodological discrepancies across the included RCTs may be responsible for the high inconsistency. Differences in the age, sex, and ejection fraction of the included patients; the variety, dosage, and intervention duration of SGLT-2is; and the definition and measurement of outcome variables may all contribute to the significant heterogeneity and diminished credibility of the results. Furthermore, the insufﬁcient sample size included in a single effect size was also a significant factor in the severe imprecision and deterioration of the evidence quality. By analyzing the reasons for this phenomenon, we believe that part of the clinical data included in SRs/MAs was derived from the subgroup analysis of large RCTs, so the lack of a study population for some specific outcomes also became an important factor affecting the quality of evidence. Regarding the decline in evidence quality due to the improper management of publication bias, according to our hypothesis, this low quality of evidence may be due to the insufficient RCTs included in the pertinent outcome measures.

Given the flawed methodology and evidence quality of the included SRs/MAs, conclusions may be biased in comparison to reality. Caution should be exercised when recommending SGLT-2is for HFpEF. Therefore, it is necessary to reintegrate and evaluate the existing evidence.

### The reestimation of outcome indicators

The high level of overlap between the included SRs/MAs means that they could not be considered independent and ideal sets of evidence, although excess significance tests indicated that there was no bias. Therefore, extracting relevant data from the primary RCTs and reestimating controversial outcome indicators is helpful to avoid the errors caused by overlap and obtain higher-quality evidence-based conclusions (29). Our updated MA showed that SGLT-2is had significant effects on first HHF, total HHF, composite of HHF or CVD, 6MWD, KCCQ-TSS, and adverse events in patients with HFpEF but did not significantly affect CVD, all-cause death, NT-proBNP level, or BNP level. The results were consistent across subgroups of composite of HHF or CVD, first HHF, total HHF, CVD, all-cause death, and 6MWD. However, the impact of SGLT-2is on NT-proBNP levels showed improvement in participants with a diagnosis of T2D or CKD. The impacts of SGLT-2is on adverse events appeared to be negative in participants with a diagnosis of T2D compared with the control group. Egger's test and sensitivity analysis indicated the stability of the results.

### Implications for further study

Our umbrella review may serve as a valuable reference for future research in the following three aspects.

First, regarding the SR/MA methodology, researchers should register research protocols promptly to ensure rigorous research procedures. Regarding the literature search strategy, we should pay attention to the grey literature retrieval method and the excluded literature list to ensure the comprehensiveness of the search and the reproducibility of the research. Before quantitative analysis, the heterogeneity of the included studies should be evaluated, and its influence on the outcome should be mitigated using subgroup analysis, meta-regression, and other techniques. To ensure the stability of the results, quantitative calculations of effect size should focus on excluding the results of a single study and analyzing the sensitivity of the included study. In addition, funnel plots, Egger's test, Begg's test, and other methods were used to evaluate publication bias, which also contributed to improving the accuracy of the MA results.

Second, a large number of RCTs specifically focused on treating HFpEF with SGLT-2is need to be implemented to avoid the problem of insufficient study samples and low evidence quality due to the inclusion of subgroup analysis. In addition, various comorbidities of patients with HFpEF also need to be fully considered when designing RCTs to clarify the efficacy of SGLT-2is in different populations. Given the available evidence, SGLT-2is can significantly reduce the incidence of HHF and adverse events as well as improve activity tolerance and quality of life in patients with HFpEF, but they do not significantly reduce the incidence of CVD and all-cause death. Future RCTs should focus on CVD and all-cause death and supplement the indicators of the impacts of SGLT-2is on the cardiac function, structure, and serum biochemistry of patients with HFpEF, thereby laying the foundation for pharmacological research. In addition, given the curative efficacy of SGLT-2is for patients with HFpEF, the design of placebo controls or blank controls should be limited in future RCTs, and for ongoing RCTS, such as “EMPAGUM research” (74), “SGLT2 Inhibitors, Ketones, and Cardiovascular Benefit Research Plan” (75), and “Sotagliflozin in Heart Failure With Preserved Ejection Fraction (HFpEF) Patients” (76), modification of the study protocol should be considered to prevent any potential ethical problems.

Finally, significant deficiencies were found in all aspects of the SR/MA report, which may be due to researchers' unfamiliarity with the relevant tools, such as AMSTAR-2, ROBIS, PRISMA, and GRADE. Therefore, evidence-based medicine education should be popularized in universities. Especially, the Cochrane Handbook and several generally recognized scales, such as AMSTAR-2, ROBIS, PRISMA, and GRADE, should be employed in these studies.

### Strengths and limitations

Our umbrella review is the first to use the AMSTAR-2, ROBIS, PRISMA guidelines, GRADE framework, CCA, and excess significance tests to summarize and assess SRs/MAs with respect to the efficacy of SGLT-2is on HFpEF. Based on our updated MA, SGLT-2is may represent an effective therapy for HFpEF by reducing HHF and adverse events and improving 6MWD and KCCQ-TSS levels. In addition, the assessment procedure showed evident limits of the present relevant SRs/MAs and RCTs, which may improve the quality of future clinical studies. However, this study is subjective concerning methodological evaluation. Although our assessment was analysed and reviewed by separate researchers, different researchers may have their unique perspectives on each item, resulting in variable outcomes.

## Conclusion

According to the available evidence, SGLT-2is appear to have a beneficial impact on HFpEF while maintaining a high level of security. Concerning the low methodological quality, reporting quality, evidence quality, and high risk of bias of the SRs/MAs supporting these findings, we should carefully draw this conclusion. Therefore, more rigorous, standardized, and comprehensive SRs/MAs are needed in related fields. More importantly, we must pay attention to the outcomes of recently updated, prospective randomized controlled, double-blind clinical trials with rigorous design and proper conduct, since they contain the least amount of bias and provide the highest level of evidence.

## Data Availability

The original contributions presented in the study are included in the article/**Supplementary Material**, further inquiries can be directed to the corresponding author.
