# Supplementary figures and images for: Scientific evidence of sodium-glucose cotransporter-2 inhibitors for heart failure with preserved ejection fraction: an umbrella review of systematic reviews and meta-analyses
Source: Front Cardiovasc Med. 2023 May 12;10:1143658. doi: 10.3389/fcvm.2023.1143658 (PMC10213331; doi:10.3389/fcvm.2023.1143658)

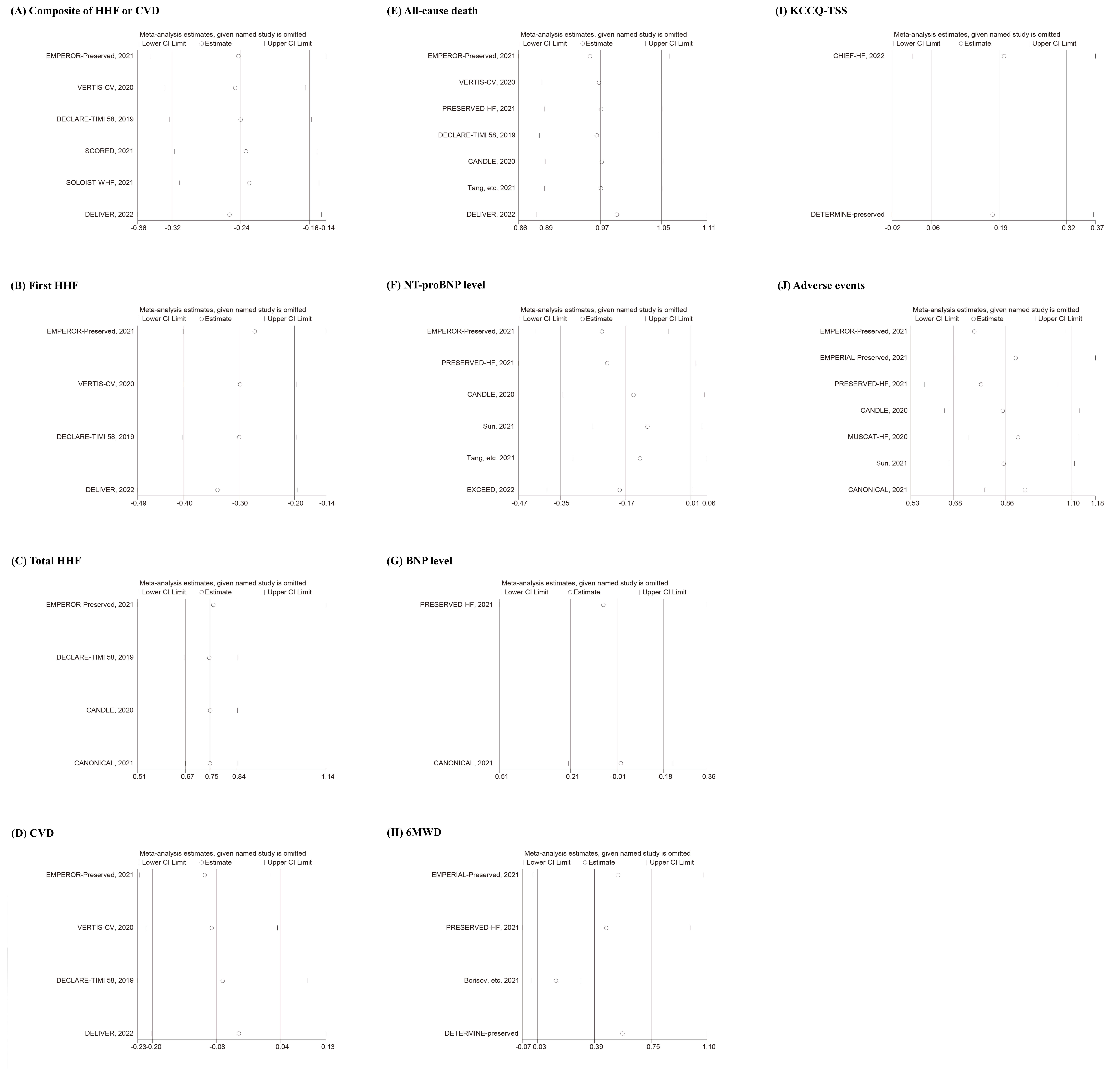

Supplement: Supplementary file 1 [file Image1.tif]
