# Supplementary material for: Scientific evidence of sodium-glucose cotransporter-2 inhibitors for heart failure with preserved ejection fraction: an umbrella review of systematic reviews and meta-analyses
Source: Front Cardiovasc Med. 2023 May 12;10:1143658. doi: 10.3389/fcvm.2023.1143658 (PMC10213331; doi:10.3389/fcvm.2023.1143658)
Supplement: Supplementary file 2 [file Table1.docx]

**Supplementary Table 1: Search strategy from database inception to December 31, 2022, for SRs/MAs of RCTs.**

| **Database** | **Search term** | **Results** |
| --- | --- | --- |
| PubMed | ((((((((Meta Analysis as Topic) OR (Data Pooling)) OR (Data Poolings)) OR (meta-analysis)) OR (meta analysis)) OR (meta-analyses)) OR (Systematic review)) OR (review, systematic)) OR (systematic reviews) **AND** ((((((((((((((Sodium-Glucose Transporter 2 Inhibitors) OR (Sodium Glucose Transporter 2 Inhibitors)) OR (SGLT-2 Inhibitors)) OR (SGLT 2 Inhibitors)) OR (SGLT2 Inhibitors)) OR (Sodium-Glucose Transporter 2 Inhibitor)) OR (Sodium Glucose Transporter 2 Inhibitor)) OR (SGLT2 Inhibitor)) OR (Inhibitor, SGLT2)) OR (Gliflozins)) OR (Gliflozin)) OR (SGLT-2 Inhibitor)) OR (Inhibitor, SGLT-2)) OR (SGLT 2 Inhibitor))) **AND** (((((Heart Failure, Diastolic) OR (Diastolic Heart Failures)) OR (Heart Failure, Preserved Ejection Fraction)) OR (Heart Failure, Normal Ejection Fraction)) OR (Diastolic Heart Failure)) | 42 |
| EMBASE | ('heart failure with preserved ejection fraction'/exp OR ('heart failure, preserved ejection fraction':ti,ab,kw OR 'diastolic heart failures':ti,ab,kw OR 'heart failure, normal ejection fraction':ti,ab,kw OR 'diastolic heart failure':ti,ab,kw)) **AND** ('sodium glucose cotransporter 2 inhibitor'/exp OR ('sodium-glucose transporter 2 inhibitors':ti,ab,kw OR 'inhibitor, sglt2':ti,ab,kw OR 'sglt-2 inhibitors':ti,ab,kw OR gliflozins:ti,ab,kw)) **AND** (('meta analysis (topic)'/exp OR ('meta analysis as topic':ti,ab,kw OR 'data poolings':ti,ab,kw OR 'meta analysis':ti,ab,kw OR 'meta analyses':ti,ab,kw)) OR ('systematic review (topic)'/exp OR ('systematic review':ti,ab,kw OR 'review, systematic':ti,ab,kw OR 'systematic reviews':ti,ab,kw))) | 47 |
| Cochrane Library | **#1** MeSH descriptor: [Heart Failure, Diastolic] this term only **#2** (Diastolic Heart Failures):ti,ab,kw OR (Heart Failure, Preserved Ejection Fraction):ti,ab,kw OR (Heart Failure, Normal Ejection Fraction):ti,ab,kw OR (Diastolic Heart Failure):ti,ab,kw OR (Heart Failure, Diastolic):ti,ab,kw (Word variations have been searched) **#3** #1 or #2 **#4** MeSH descriptor: [Sodium-Glucose Transporter 2 Inhibitors] this term only **#5** (Sodium Glucose Transporter 2 Inhibitors):ti,ab,kw OR (SGLT-2 Inhibitors):ti,ab,kw OR (Inhibitor, SGLT2):ti,ab,kw OR (Gliflozins):ti,ab,kw OR (Inhibitor, SGLT-2):ti,ab,kw **#6** #4 or #5 **#7** MeSH descriptor: [Meta-Analysis as Topic] this term only **#8** (Data Poolings):ti,ab,kw OR (meta-analysis):ti,ab,kw OR (meta-analyses):ti,ab,kw **#9** #7 or #8 **#10** MeSH descriptor: [Systematic Reviews as Topic] this term only **#11** (Systematic review):ti,ab,kw OR (review, systematic):ti,ab,kw OR (systematic reviews):ti,ab,kw **#12** #10 or #11 **#13** #9 or #12 **#14** #3 and #6 #13 | 2 |
| Other sources | (NCT03753087) OR (Effects of Empagliflozin on Exercise Capacity and Left Ventricular Diastolic Function in Patients With Heart Failure With Preserved Ejection Fraction and Type-2 Diabetes Mellitus);  (NCT03030235) OR (PRESERVED-HF);  (NCT03619213) OR (DELIVER);  (NCT03877224) OR (DETERMINE-preserved);  (NCT04730947) OR ((Dapagliflozin (DAPA) Effects in HFpEF));  (NCT03057951) OR (EMPEROR-Preserved);  (NCT03448406) OR ((This Study Tests Empagliflozin in Patients With Chronic Heart Failure With Preserved Ejection Fraction (HFpEF). The Study Looks at How Far Patients Can Walk in 6 Minutes and at Their Heart Failure Symptoms)) | 36 |
| Total |  | 127 |

**Notes:** RCTs: randomized controlled trials; SRs/MAs: systematic reviews and meta-analyses; SGLT-2 Inhibitors: Sodium-Glucose Transporter 2 Inhibitors.
