# Supplementary material for: Scientific evidence of sodium-glucose cotransporter-2 inhibitors for heart failure with preserved ejection fraction: an umbrella review of systematic reviews and meta-analyses
Source: Front Cardiovasc Med. 2023 May 12;10:1143658. doi: 10.3389/fcvm.2023.1143658 (PMC10213331; doi:10.3389/fcvm.2023.1143658)
Supplement: Supplementary file 3 [file Table2.docx]

**Supplementary Table 2: Detailed information on excluded literature.**

| **Title** | **Author** | **Year** | **Reasons for exclusion** |
| --- | --- | --- | --- |
| Management of Noncardiac Comorbidities in Chronic Heart Failure. | V. H. Chong, et al | 2015 | The title and abstract were not consistent with our research topic. |
| Reframing the association and significance of co-morbidities in heart failure. | F. Triposkiadis, et al | 2016 | The title and abstract were not consistent with our research topic. |
| Cardiometabolic Syndrome and Increased Risk of Heart Failure. | H. von Bibra, et al | 2016 | The title and abstract were not consistent with our research topic. |
| Editorial commentary: Anti-glycemic drugs and heart failure: A new era. | D. Fitchett, et al | 2017 | The title and abstract were not consistent with our research topic. |
| Comparison of costs and outcomes of dapagliflozin with other glucose-lowering therapy classes added to metformin using a short-term cost-effectiveness model in the US setting | A. Chakravarty, et al | 2018 | The title and abstract were not consistent with our research topic. |
| Preventing Heart Failure in Diabetes: Glycemic Targets or Class Effect? | A. D. DeVore, et al | 2018 | The title and abstract were not consistent with our research topic. |
| Insulin and glucose-lowering agents for treating people with diabetes and chronic kidney disease. | C. Lo, et al | 2018 | The title and abstract were not consistent with our research topic. |
| December 2018 at a glance: new imaging methods, translational cardiology and non-medical treatment. | M. Metra, et al | 2018 | The title and abstract were not consistent with our research topic. |
| Effect of Dapagliflozin on the Progression From Prediabetes to T2DM in Subjects With Myocardial Infarction. | J. Al-Suwaidi, et al | 2018 | The title and abstract were not consistent with our research topic. |
| Effects of sodium glucose cotransporter type 2 inhibitors on heart failure. | M. E. Nassif, et al | 2019 | The title and abstract were not consistent with our research topic. |
| Highlights in heart failure. | D. Tomasoni, et al | 2019 | The title and abstract were not consistent with our research topic. |
| Harms and benefits of sodium-glucose co-transporter 2 inhibitors. | T. Chesterman, et al | 2020 | The title and abstract were not consistent with our research topic. |
| Trends in prevalence of comorbidities in heart failure clinical trials. | M. S. Khan, et al | 2020 | The title and abstract were not consistent with our research topic. |
| SGLT2 inhibitors and atrial fibrillation in type 2 diabetes: a systematic review with meta-analysis of 16 randomized controlled trials. | W. J. Li, et al | 2020 | The title and abstract were not consistent with our research topic. |
| Design of a prospective patient-level pooled analysis of two parallel trials of empagliflozin in patients with established heart failure. | M. Packer, et al | 2020 | The title and abstract were not consistent with our research topic. |
| Updated Meta-analysis Assessing the Effect of Sodium-Glucose Co-transporter-2 Inhibitors on Surrogate End points in Patients With Heart Failure With Reduced Ejection Fraction. | D. Patoulias, et al | 2020 | The title and abstract were not consistent with our research topic. |
| Effects of antidiabetic drugs on left ventricular function/dysfunction: a systematic review and network meta-analysis. | D. P. Zhang, et al | 2020 | The title and abstract were not consistent with our research topic. |
| Sodium-glucose co-transporter-2 inhibitors and major adverse limb events: A trial-level meta-analysis including 51 713 individuals | C. Y. Huang, et al | 2020 | The title and abstract were not consistent with our research topic. |
| Dipeptidyl peptidase-4 inhibitors glucagon-like peptide 1 receptor agonists and sodium-glucose co-transporter-2 inhibitors for people with cardiovascular disease: a network meta-analysis | T. Kanie, et al | 2021 | The title and abstract were not consistent with our research topic. |
| The safety outcomes of sodium-glucose cotransporter 2 inhibitors in patients with different renal function: A systematic review and meta-analysis | Y. Bai, et al | 2021 | The title and abstract were not consistent with our research topic. |
| SGLT2 inhibitors and cardiac remodelling: a systematic review and meta-analysis of randomized cardiac magnetic resonance imaging trials. | N. K. Dhingra, et al | 2021 | The title and abstract were not consistent with our research topic. |
| The Evolution of Sodium-Glucose Co-Transporter-2 Inhibitors in Heart Failure. | O. Fadiran, et al | 2021 | The title and abstract were not consistent with our research topic. |
| Sodium-glucose cotransporter 2 inhibitors in heart failure with preserved ejection fraction: A protocol for meta-analysis. | H. Fukuta, et al | 2021 | The title and abstract were not consistent with our research topic. |
| Obesity and Heart Failure with Preserved Ejection Fraction. | E. Koutroumpakis, et al | 2021 | The title and abstract were not consistent with our research topic. |
| An evaluation of canagliflozin for the treatment of type 2 diabetes: an update. | T. Minami, et al | 2021 | The title and abstract were not consistent with our research topic. |
| Meta-Analysis Assessing the Cardiovascular Efficacy of Sodium-Glucose Co-Transporter-2 Inhibitors According to Baseline Treatment of Interest. | D. Patoulias, et al | 2021 | The title and abstract were not consistent with our research topic. |
| Sodium-glucose cotransporter 2 inhibitor effects on heart failure hospitalization and cardiac function: systematic review. | R. Rasalam, et al | 2021 | The title and abstract were not consistent with our research topic. |
| Letter by Tanaka and Node Regarding Article, "Type 2 Diabetes Mellitus and Impact of Heart Failure on Prognosis Compared to Other Cardiovascular Diseases: A Nationwide Study. | A. Tanaka, et al | 2021 | The title and abstract were not consistent with our research topic. |
| Natriuretic Peptide–Based Risk Prediction and Assessment of Treatment Effect: Revisited in This Era. | A. Tanaka, et al | 2021 | The title and abstract were not consistent with our research topic. |
| Effect of sodium-glucose cotransporter 2 inhibitors on cardiac structure and function in type 2 diabetes mellitus patients with or without chronic heart failure: a meta-analysis. | Y. W. Yu, et al | 2021 | The title and abstract were not consistent with our research topic. |
| Effects of Sodium/Glucose Cotransporter 2 (SGLT2) Inhibitors on Cardiovascular and Metabolic Outcomes in Patients Without Diabetes Mellitus: A Systematic Review and Meta-Analysis of Randomized-Controlled Trials. | Y. H. Teo, et al | 2021 | The title and abstract were not consistent with our research topic. |
| Left bundle branch pacing for cardiac resynchronization therapy: A systematic literature review and meta-analysis | C. Zhong, et al | 2021 | The title and abstract were not consistent with our research topic. |
| Meta-analysis of the Usefulness of Catheter Ablation of Atrial Fibrillation in Patients With Heart Failure With Preserved Ejection Fraction | O. M. Aldaas, et al | 2021 | The title and abstract were not consistent with our research topic. |
| A heart failure phenotype stratified model for predicting 1-year mortality in patients admitted with acute heart failure: results from an individual participant data meta-analysis of four prospective European cohorts | Y. Chen, et al | 2021 | The title and abstract were not consistent with our research topic. |
| The Evolution of Sodium-Glucose Co-Transporter-2 Inhibitors in Heart Failure | O. Fadiran, et al | 2021 | The title and abstract were not consistent with our research topic. |
| Association between evidence-based medication at discharge and outcomes in patients with heart failure: a systematic review and meta-analysis | J. L. Feng, et al | 2021 | The title and abstract were not consistent with our research topic. |
| Sodium-glucose cotransporter 2 inhibitors in heart failure with preserved ejection fraction: A protocol for meta-analysis | H. Fukuta, et al | 2021 | The title and abstract were not consistent with our research topic. |
| Iron replacement therapy in heart failure: a literature review | H. Ismahel, et al | 2021 | The title and abstract were not consistent with our research topic. |
| Comparison of Pharmacological Treatment Effects on Long-Time Outcomes in Heart Failure With Preserved Ejection Fraction: A Network Meta-analysis of Randomized Controlled Trials | Y. Lin, et al | 2021 | The title and abstract were not consistent with our research topic. |
| Beta-blockers and inhibitors of the renin-angiotensin aldosterone system for chronic heart failure with preserved ejection fraction | N. Martin, et al | 2021 | The title and abstract were not consistent with our research topic. |
| Impact of oral soluble guanylate cyclase stimulators in heart failure: A systematic review and Meta-analysis of randomized controlled trials | N. Moghaddam, et al | 2021 | The title and abstract were not consistent with our research topic. |
| The Effect of Sacubitril-Valsartan in Heart Failure Patients With Mid-Range and Preserved Ejection Fraction: A Meta-Analysis | D. Nie, et al | 2021 | The title and abstract were not consistent with our research topic. |
| A new approach to the clinical subclassification of heart failure with preserved ejection fraction | H. Nouraei, et al | 2021 | The title and abstract were not consistent with our research topic. |
| Angiotensin converting enzyme inhibitors and angiotensin II receptor blockers and outcomes in patients with acute decompensated heart failure: a systematic review and meta-analysis | J. Pelayo, et al | 2021 | The title and abstract were not consistent with our research topic. |
| Diagnosing heart failure in primary care: individual patient data meta-analysis of two European prospective studies | A. K. Roalfe, et al | 2021 | The title and abstract were not consistent with our research topic. |
| Cardiac and Noncardiac Disease Burden and Treatment Effect of Sacubitril/Valsartan: Insights From a Combined PARAGON-HF and PARADIGM-HF Analysis | L. E. Rohde, et al | 2021 | The title and abstract were not consistent with our research topic. |
| Effects of angiotensin receptor neprilysin inhibitor on renal function in patients with heart failure: a systematic review and meta-analysis | Y. Shi, et al | 2021 | The title and abstract were not consistent with our research topic. |
| Age dependent associations of risk factors with heart failure: pooled population based cohort study | J. Tromp, et al | 2021 | The title and abstract were not consistent with our research topic. |
| Worsening Heart Failure Episodes Outside a Hospital Setting in Heart Failure With Preserved Ejection Fraction: The PARAGON-HF Trial | M. Vaduganathan, et al | 2021 | The title and abstract were not consistent with our research topic. |
| Effect of Angiotensin-Neprilysin Versus Renin-Angiotensin System Inhibition on Renal Outcomes: A Systematic Review and Meta-Analysis | Y. Xu, et al | 2021 | The title and abstract were not consistent with our research topic. |
| Effects of sodium-glucose cotransporter type 2 inhibitors on cardiovascular | C. Zheng, et al | 2021 | The title and abstract were not consistent with our research topic. |
| The Effectiveness of Sodium-Glucose Cotransporter 2 Inhibitors and Glucagon-like Peptide-1 Receptor Agonists on Cardiorenal Outcomes: Systematic Review and Meta-analysis. | M. U. Ali, et al | 2022 | The title and abstract were not consistent with our research topic. |
| Polypharmacy definition and prevalence in heart failure: a systematic review. | J. Beezer, et al | 2022 | The title and abstract were not consistent with our research topic. |
| Chronic heart failure: epidemiology, investigation and management. | S. A. S. Beggs, et al | 2022 | The title and abstract were not consistent with our research topic. |
| Current Understanding of Molecular Pathophysiology of Heart Failure with Preserved Ejection Fraction. | H. Budde, et al | 2022 | The title and abstract were not consistent with our research topic. |
| Will Diabetes Drug Revolutionize Heart Failure Treatment? | R. Davidson, et al | 2022 | The title and abstract were not consistent with our research topic. |
| Benefits of sodium glucose cotransporter 2 inhibitors across the spectrum of cardiovascular diseases. | G. S. Gulsin, et al | 2022 | The title and abstract were not consistent with our research topic. |
| The dawn of a new era of targeted therapies for heart failure with preserved ejection fraction. | K. Harada, et al | 2022 | The title and abstract were not consistent with our research topic. |
| The effects of SGLT-2 inhibitors vs renin-angiotensin-aldosterone system inhibition on cardiovascular outcomes of heart failure with preserved ejection fraction: network meta-analysis. | J. Acevedo, et al | 2022 | The title and abstract were not consistent with our research topic. |
| Effect of sodium-glucose cotransporter-2 inhibitors on blood pressure in patients with heart failure: a systematic review and meta-analysis. | M. Li, et al | 2022 | The title and abstract were not consistent with our research topic. |
| Effects of SGLT2 inhibitors on cardiovascular outcomes in patients with stage 3/4 CKD: A meta-analysis. | N. Li, et al | 2022 | The title and abstract were not consistent with our research topic. |
| Sodium-glucose cotransporter 2 inhibitors: the first universal treatment for heart failure? | K. McDowell, et al | 2022 | The title and abstract were not consistent with our research topic. |
| RENAL EFFECTS OF SODIUM-GLUCOSE CO-TRANSPORTER-2 INHIBITORS IN PATIENTS WITH HEART FAILURE WITH REDUCED OR PRESERVED EJECTION FRACTION. | D. Patoulias, et al | 2022 | The title and abstract were not consistent with our research topic. |
| SGLT-2 inhibitors and cardiovascular outcomes in patients with and without a history of heart failure: a systematic review and meta-analysis. | V. Razuk, et al | 2022 | The title and abstract were not consistent with our research topic. |
| PULMONARY EMBOLISM AND HEART FAILURE FROM EXOGENOUS TESTOSTERONE USE. | A. Sachdeva, et al | 2022 | The title and abstract were not consistent with our research topic. |
| Beneficial Effect of Sodium-Glucose Co-transporter 2 Inhibitors on Left Ventricular Function. | F. H. Shi, et al | 2022 | The title and abstract were not consistent with our research topic. |
| New antidiabetic therapy and HFpEF: light at the end of tunnel? | M. Tadic, et al | 2022 | The title and abstract were not consistent with our research topic. |
| Influence of receptor selectivity on benefits from SGLT2 inhibitors in patients with heart failure: a systematic review and head-to-head comparative efficacy network meta-analysis. | T. Täger, et al | 2022 | The title and abstract were not consistent with our research topic. |
| Comparing Sacubitril/Valsartan Against Sodium-Glucose Cotransporter 2 Inhibitors in Heart Failure: A Systematic Review and Network Meta-analysis. | Y. N. Teo, et al | 2022 | The title and abstract were not consistent with our research topic. |
| The impact of SGLT2 inhibition on imaging markers of cardiac function: A systematic review and meta-analysis. | P. Theofilis, et al | 2022 | The title and abstract were not consistent with our research topic. |
| Comparison of cardiovascular outcomes and cardiometabolic risk factors between patients with type 2 diabetes treated with sodium-glucose cotransporter-2 inhibitors and dipeptidyl peptidase-4 inhibitors: a meta-analysis. | S. Wang, et al | 2022 | The title and abstract were not consistent with our research topic. |
| Effects of Sodium/Glucose Cotransporter 2. | C. F. Wee, et al | 2022 | The title and abstract were not consistent with our research topic. |
| Optimal Pharmacologic Treatment of Heart Failure with Preserved and Mildly Reduced Ejection Fraction: A Meta-analysis. | B. Xiang, et al | 2022 | The title and abstract were not consistent with our research topic. |
| Effect of sodium-glucose cotransporter-2 inhibitors on cardiac remodeling: a systematic review and meta-analysis. | N. Zhang, et al | 2022 | The title and abstract were not consistent with our research topic. |
| Effect of empagliflozin in patients with heart failure across the spectrum of left ventricular ejection fraction. | J. Butler, et al | 2022 | The title and abstract were not consistent with our research topic. |
| The Efficacy and Safety of the Combined Therapy of Sodium-Glucose Co-Transporter-2 Inhibitors and Angiotensin Receptor-Neprilysin Inhibitor in Patients With Heart Failure With Reduced Ejection Fraction: A Meta-Analysis of the EMPEROR-Reduced and DAPA-HF Sub-Analysis | Y. Lin, et al | 2022 | The title and abstract were not consistent with our research topic. |
| Benefits of SGLT2i for the Treatment of Heart Failure Irrespective of Diabetes Diagnosis: A State-of-the-Art Review | E. Delgado, et al | 2022 | The title and abstract were not consistent with our research topic. |
| Pharmacotherapies in Heart Failure With Preserved Ejection Fraction: A Systematic Review and Meta-Analysis of Randomized Controlled Trials | N. Baral, et al | 2021 | The title and abstract were not consistent with our research topic. |
| Effect of empagliflozin in patients with heart failure across the spectrum of left ventricular ejection fraction | J. Butler | 2022 | The title and abstract were not consistent with our research topic. |
| Optimal Pharmacologic Treatment of Heart Failure With Preserved and Mildly Reduced Ejection Fraction: A Meta-analysis | B. Xiang, et al | 2022 | The title and abstract were not consistent with our research topic. |
| Safety outcomes of SGLT2i in the heart failure trials: A systematic review and Meta-analysis | A. M. Younes, et al | 2022 | The title and abstract were not consistent with our research topic. |
| The survival of patients with heart failure with preserved or reduced left ventricular ejection fraction: an individual patient data meta-analysis | MAGGIC | 2012 | Lack of relevant outcome indicators. |
| Heart failure hospitalization with SGLT-2 inhibitors: a systematic review and meta-analysis of randomized controlled and observational studies | A. K. Singh, et al | 2019 | Lack of relevant outcome indicators. |
| Surrogate cardiovascular outcomes with sodium-glucose co-transporter-2 inhibitors in women: An updated meta-analysis | D. Patoulias, et al | 2021 | Lack of relevant outcome indicators. |
| Effect of Dapagliflozin, Compared With Placebo, According to Baseline Risk in DAPA-HF | K. F. Docherty, et al | 2022 | Lack of relevant outcome indicators. |
| Safety outcomes of SGLT2i in the heart failure trials: A systematic review and Meta-analysis. | A. M. Younes, et al | 2022 | Lack of relevant outcome indicators. |
| A Meta-Analysis of the Sodium-Glucose Cotransporter 2 Inhibitors in Patients with Heart Failure and Preserved Ejection Fraction. | A. Al-Abdouh, et al | 2022 | Readers’ comments |
| Meta-Analysis Assessing the Impact of Major Co-Morbidities, Gender, and Race on Cardiovascular Efficacy of Sodium-Glucose Co-Transporter-2 Inhibitors Among Patients with Heart Failure With Preserved or Reduced Ejection Fraction. | D. Patoulias, et al | 2022 | Readers’ comments |
| Sodium glucose co-transporter 2 inhibitors in heart failure with preserved ejection fraction: a systematic review and meta-analysis. | V. Tsampasian, et al | 2022 | Research letter |
