# Supplementary material for: Scientific evidence of sodium-glucose cotransporter-2 inhibitors for heart failure with preserved ejection fraction: an umbrella review of systematic reviews and meta-analyses
Source: Front Cardiovasc Med. 2023 May 12;10:1143658. doi: 10.3389/fcvm.2023.1143658 (PMC10213331; doi:10.3389/fcvm.2023.1143658)
Supplement: Supplementary file 4 [file Table3.docx]

**Supplementary Table 3: Summary of the primary RCTs included in the 15 SRs/MAs and CCA calculation process.**

| **Study ID** | **Butler J,  2020(44)** | **Lu Y,  2021(45)** | **Zheng CY,  2021(46)** | **Singh A,  2021(47)** | **Cardoso R,  2021(48)** | **Pandey A,  2022(49)** | **Vaduganathan M,  2022(50)** | **Razuk V, 2022(51)** |
| --- | --- | --- | --- | --- | --- | --- | --- | --- |
| **EMPEROR-Preserved, 2021** | NO | NO | NO | NO | NO | YES | YES | YES |
| **VERTIS-CV, 2020** | YES | NO | YES | YES | YES | NO | NO | NO |
| **EMPERIAL-Preserved, 2021** | NO | NO | NO | NO | NO | NO | NO | NO |
| **PRESERVED-HF, 2021** | NO | NO | NO | NO | NO | NO | NO | NO |
| **DECLARE-TIMI 58, 2019** | YES | YES | YES | YES | YES | NO | NO | NO |
| **SCORED, 2021** | NO | NO | NO | YES | YES | NO | NO | YES |
| **CANDLE, 2020** | NO | NO | NO | NO | NO | NO | NO | NO |
| **MUSCAT-HF, 2020** | NO | NO | NO | NO | NO | NO | NO | NO |
| **Borisov, etc. 2021** | NO | NO | NO | NO | NO | NO | NO | NO |
| **Sun. 2021** | NO | NO | NO | NO | NO | NO | NO | NO |
| **TANG, etc. 2021** | NO | NO | NO | NO | NO | NO | NO | NO |
| **SOLOIST-WHF, 2021** | YES | YES | NO | YES | YES | YES | NO | YES |
| **CANONICAL, 2021** | NO | NO | NO | NO | NO | NO | NO | NO |
| **DELIVER, 2022** | NO | NO | NO | NO | NO | NO | YES | NO |
| **EXCEED, 2022** | NO | NO | NO | NO | NO | NO | NO | NO |
| **CHIEF-HF, 2022** | NO | NO | NO | NO | NO | NO | NO | NO |
| **DETERMINE-preserved,  No publication** | NO | NO | NO | NO | NO | NO | NO | NO |
| **Total RCTs included** | **3** | **2** | **2** | **4** | **4** | **2** | **2** | **3** |

**Supplementary Table 3: continue.**

| **Study ID** | **Cao Y,  2022(52)** | **Zhao LY,  2022(53)** | **Yang DN,  2022(54)** | **Fukuta H,  2022(55)** | **Zhou HF,  2022(56)** | **Jhund PS, 2022 (57)*** | **Wang YT, 2022(58)** | **Number of times included** |
| --- | --- | --- | --- | --- | --- | --- | --- | --- |
| **EMPEROR-Preserved, 2021** | YES | YES | YES | YES | YES | NO | YES | **9** |
| **VERTIS-CV, 2020** | YES | YES | YES | YES | YES | NO | YES | **10** |
| **EMPERIAL-Preserved, 2021** | NO | NO | YES | YES | YES | NO | NO | **3** |
| **PRESERVED-HF, 2021** | NO | YES | YES | YES | YES | NO | NO | **4** |
| **DECLARE-TIMI 58, 2019** | YES | YES | YES | YES | YES | NO | YES | **11** |
| **SCORED, 2021** | YES | YES | YES | YES | YES | NO | YES | **9** |
| **CANDLE, 2020** | NO | NO | NO | YES | YES | NO | NO | **2** |
| **MUSCAT-HF, 2020** | NO | NO | NO | YES | YES | NO | NO | **2** |
| **Borisov, etc. 2021** | NO | NO | NO | NO | YES | NO | NO | **1** |
| **Sun. 2021** | NO | NO | NO | NO | YES | NO | NO | **1** |
| **TANG, etc. 2021** | NO | NO | YES | NO | NO | NO | NO | **1** |
| **SOLOIST-WHF, 2021** | YES | YES | YES | YES | YES | NO | YES | **12** |
| **CANONICAL, 2021** | NO | NO | NO | YES | YES | NO | NO | **2** |
| **DELIVER, 2022** | NO | NO | NO | NO | NO | YES | YES | **3** |
| **EXCEED, 2022** | NO | NO | NO | YES | NO | NO | NO | **1** |
| **CHIEF-HF, 2022** | NO | NO | YES | NO | NO | NO | NO | **1** |
| **DETERMINE-preserved,  No publication** | NO | NO | YES | NO | NO | NO | NO | **1** |
| **Total RCTs included** | **5** | **6** | **10** | **11** | **12** | **1** | **6** | **CCA = 23.53%** |

**Notes:** * RCTs included in **Jhund PS, 2022** involving both HFrEF and HFpEF, and there was only one RCT (DELIVER) associated with HFpEF. SRs/MAs: systematic reviews and meta-analyses; RCTs: randomized controlled trials; CCA: corrected covered area. The tabular data are presented in columns corresponding to a total of 17 unique primary RCTs and rows corresponding to the 15 SRs/MAs. “YES” indicate when a primary RCT is included in an SR/MA. The total number of primary RCTs included in each SR/MA is presented in the last row. The total number of times a primary RCT is included in SRs/MAs is presented in the last columns. The total number of RCTs included in SRs/MAs is taken as "*N*" (repetition allowed), the total number of RCTs is "*r*" and the number of included SRs/MAs is "*c*", CCA= (*N* - *r*) / [(*r* × *c*) – *r*]. *N* = 73, *r* = 17, *c* = 15.
