# Supplementary material for: Scientific evidence of sodium-glucose cotransporter-2 inhibitors for heart failure with preserved ejection fraction: an umbrella review of systematic reviews and meta-analyses
Source: Front Cardiovasc Med. 2023 May 12;10:1143658. doi: 10.3389/fcvm.2023.1143658 (PMC10213331; doi:10.3389/fcvm.2023.1143658)
Supplement: Supplementary file 5 [file Table4.docx]

**Supplementary Table 4: Results of the ROBIS assessments.**

| **Author, year (Country)** | **Phase 1** | **Phase 2** | | | | **Phase 3** |  |
| --- | --- | --- | --- | --- | --- | --- | --- |
|  | Assessing relevance | Domain 1: study eligibility criteria | Domain 2: identification and selection of studies | Domain 3: collection  and study appraisal | Domain 4: synthesis  and findings | Risk of bias in  the review |  |
| **Butler J, 2020 (USA) (44)** | | √ | √ | √ | √ | × | × |
| **Lu Y, 2021 (CHN) (45)** | | √ | √ | √ | × | × | √ |
| **Zheng CY, 2021 (CHN) (46)** | | √ | √ | √ | √ | × | √ |
| **Singh A, 2021 (IND) (47)** | | √ | √ | × | √ | × | √ |
| **Cardoso R, 2021 (USA) (48)** | | √ | √ | √ | √ | × | √ |
| **Pandey A, 2022 (CNA) (49)** | | √ | √ | √ | √ | × | × |
| **Vaduganathan M, 2022 (USA) (50)** | | √ | √ | √ | √ | × | × |
| **Razuk V, 2022 (USA) (51)** | | √ | √ | × | √ | × | √ |
| **Cao Y, 2022 (CHN) (52)** | | √ | √ | √ | × | × | √ |
| **Zhao LY, 2022 (CHN) (53)** | | √ | √ | √ | √ | × | √ |
| **Yang DN, 2022 (CHN) (54)** | | √ | √ | √ | × | × | × |
| **Fukuta H, 2022 (JPN) (55)** | | √ | √ | √ | √ | × | × |
| **Zhou HF, 2022 (CHN) (56)** | | √ | √ | √ | √ | √ | √ |
| **Jhund PS, 2022 (UK) (57)** | | √ | √ | × | × | × | √ |
| **Wang YT, 2022 (CHN) (58)** | | √ | √ | √ | √ | × | √ |

**Notes:** √: low risk; ×: high risk. CHN: China; CAN: Canada; IND: India; JPN: Japan; UK: United Kingdom; USA: United States of America.
