# Supplementary material for: Scientific evidence of sodium-glucose cotransporter-2 inhibitors for heart failure with preserved ejection fraction: an umbrella review of systematic reviews and meta-analyses
Source: Front Cardiovasc Med. 2023 May 12;10:1143658. doi: 10.3389/fcvm.2023.1143658 (PMC10213331; doi:10.3389/fcvm.2023.1143658)
Supplement: Supplementary file 6 [file Table5.docx]

**Supplementary Table 5: Results of the PRISMA checklist.**

| **Section and topic** | | **Items** | **Butler J (44)** | **Lu**  **Y (45)** | **Zheng**  **CY (46)** | **Singh A (47)** | **Cardoso R (48)** | **Pandey**  **A (49)** | **Vaduganathan M (50)** | **Razuk**  **V (51)** | **Cao**  **Y (52)** | **Zhao**  **LY (53)** | **Yang**  **DN (54)** | **Fukuta H (55)** | **Zhou**  **HF (56)** | **Jhund PS (57)** | **Wang**  **YT (58)** | **The percentage  of "Y" (%)** |
| --- | --- | --- | --- | --- | --- | --- | --- | --- | --- | --- | --- | --- | --- | --- | --- | --- | --- | --- |
| Title | Title | 1 | Y | Y | Y | Y | Y | Y | Y | Y | Y | Y | Y | Y | Y | Y | Y | 100.00% |
| Abstract | Abstracts checklist | 2 | N | N | N | N | N | N | Y | Y | N | N | N | Y | Y | N | Y | 33.33% |
| Introduction | Rationale | 3 | Y | Y | Y | Y | Y | Y | Y | Y | Y | Y | Y | Y | Y | Y | Y | 100.00% |
|  | Objectives | 4 | Y | Y | Y | Y | Y | Y | Y | Y | Y | Y | Y | Y | Y | Y | Y | 100.00% |
| Methods | Eligibility criteria | 5 | Y | Y | Y | Y | Y | Y | Y | Y | Y | Y | Y | Y | Y | N | Y | 93.33% |
|  | Information sources | 6 | Y | Y | Y | Y | Y | Y | Y | Y | Y | Y | Y | Y | Y | N | Y | 93.33% |
|  | Search strategy | 7 | Y | Y | Y | N | Y | Y | Y | N | Y | Y | Y | Y | Y | N | Y | 80.00% |
|  | Selection process | 8 | Y | Y | Y | Y | Y | Y | Y | Y | Y | Y | Y | Y | Y | N | Y | 93.33% |
|  | Data collection process | 9 | Y | Y | Y | Y | Y | Y | Y | Y | Y | Y | Y | Y | Y | N | Y | 93.33% |
|  | Data items | 10 (a) | Y | Y | Y | Y | Y | Y | Y | Y | Y | Y | Y | Y | Y | Y | Y | 100.00% |
|  |  | 10 (b) | Y | Y | Y | Y | Y | Y | Y | Y | Y | Y | Y | Y | Y | Y | Y | 100.00% |
|  | Study risk of bias assessment | 11 | Y | Y | Y | Y | Y | Y | Y | Y | Y | Y | Y | Y | Y | N | Y | 93.33% |
|  | Effect measures | 12 | Y | Y | Y | Y | Y | Y | Y | Y | Y | Y | Y | Y | Y | Y | Y | 100.00% |
|  | Synthesis methods | 13 (a) | Y | Y | Y | Y | Y | Y | Y | Y | Y | Y | Y | Y | Y | N | Y | 93.33% |
|  |  | 13 (b) | Y | Y | Y | Y | Y | Y | Y | Y | Y | Y | Y | Y | Y | Y | Y | 100.00% |
|  |  | 13 (c) | Y | Y | Y | Y | Y | Y | Y | Y | Y | Y | Y | Y | Y | Y | Y | 100.00% |
|  |  | 13 (d) | Y | Y | Y | Y | Y | Y | Y | Y | Y | Y | Y | Y | Y | Y | Y | 100.00% |
|  |  | 13 (e) | N | Y | Y | Y | Y | N | N | Y | Y | Y | N | N | Y | Y | Y | 66.67% |
|  |  | 13 (f) | Y | Y | Y | Y | N | Y | N | N | Y | Y | Y | Y | Y | Y | Y | 80.00% |
|  | Reporting bias assessment | 14 | N | Y | Y | Y | Y | N | N | Y | Y | Y | N | N | Y | N | N | 53.33% |
|  | Certainty assessment | 15 | N | N | N | N | N | N | N | N | N | N | Y | N | Y | Y | N | 20.00% |
| Result | Study selection | 16 (a) | Y | Y | Y | Y | Y | Y | Y | Y | Y | Y | Y | Y | Y | N | Y | 93.33% |
|  |  | 16 (b) | N | N | N | N | N | N | N | N | N | N | N | N | Y | N | N | 6.67% |
|  | Study characteristics | 17 | Y | Y | Y | Y | Y | Y | Y | Y | Y | Y | Y | Y | Y | Y | Y | 100.00% |
|  | Risk of bias in studies | 18 | Y | Y | Y | Y | Y | Y | Y | Y | Y | Y | Y | Y | Y | N | Y | 93.33% |
|  | Results of individual studies | 19 | Y | Y | Y | Y | Y | Y | Y | Y | Y | Y | Y | Y | Y | Y | Y | 100.00% |
|  | Results of syntheses | 20 (a) | Y | Y | Y | Y | Y | Y | Y | Y | Y | Y | Y | Y | Y | Y | Y | 100.00% |
|  |  | 20 (b) | Y | Y | Y | Y | Y | Y | Y | Y | Y | Y | Y | Y | Y | Y | Y | 100.00% |
|  |  | 20 (c) | N | Y | Y | Y | Y | N | N | Y | Y | Y | N | N | Y | Y | Y | 66.67% |
|  |  | 20 (d) | Y | Y | Y | Y | N | Y | N | N | Y | Y | Y | Y | Y | Y | Y | 80.00% |
|  | Reporting biases | 21 | N | Y | Y | Y | Y | N | N | Y | Y | Y | N | N | Y | N | N | 53.33% |
|  | Certainty of evidence | 22 | N | N | N | N | N | N | N | N | N | N | Y | N | Y | N | N | 13.33% |
| Discussion | Discussion | 23 (a) | Y | Y | Y | Y | Y | Y | Y | Y | Y | Y | Y | Y | Y | Y | Y | 100.00% |
|  |  | 23 (b) | Y | Y | Y | Y | Y | Y | Y | Y | Y | Y | Y | Y | Y | Y | Y | 100.00% |
|  |  | 23 (c) | Y | Y | Y | Y | Y | Y | Y | Y | Y | Y | Y | Y | Y | Y | Y | 100.00% |
|  |  | 23 (d) | Y | Y | Y | Y | Y | Y | Y | Y | Y | Y | Y | Y | Y | Y | Y | 100.00% |
| Other information | Registration and protocol | 24 (a) | N | N | N | N | N | N | Y | Y | N | N | N | Y | Y | N | Y | 33.33% |
|  |  | 24(b) | N | N | N | N | N | N | Y | Y | N | N | N | Y | Y | N | Y | 33.33% |
|  |  | 24 (c) | N | N | N | N | N | N | N | N | N | N | N | N | N | N | N | 0.00% |
|  | Support | 25 | Y | Y | Y | Y | Y | Y | Y | Y | Y | Y | Y | Y | Y | Y | Y | 100.00% |
|  | Competing interests | 26 | Y | Y | Y | Y | Y | Y | Y | Y | Y | Y | Y | Y | Y | Y | Y | 100.00% |
|  | Availability of data,  code and other materials | 27 | Y | Y | Y | Y | Y | Y | Y | Y | Y | Y | Y | Y | Y | Y | Y | 100.00% |

**Notes:** Y: yes; N: no.
