# Supplementary material for: Scientific evidence of sodium-glucose cotransporter-2 inhibitors for heart failure with preserved ejection fraction: an umbrella review of systematic reviews and meta-analyses
Source: Front Cardiovasc Med. 2023 May 12;10:1143658. doi: 10.3389/fcvm.2023.1143658 (PMC10213331; doi:10.3389/fcvm.2023.1143658)
Supplement: Supplementary file 7 [file Table6.docx]

**Supplementary Table 6: Results of Egger’s test.**

|  | **Coefficient** | **Standard error** | ***P*** |
| --- | --- | --- | --- |
| **Composite of HHF or CVD** | -0.71 | 0.69 | 0.36 |
| **First HHF** | -0.24 | 0.54 | 0.70 |
| **Total HHF** | -0.07 | 0.22 | 0.78 |
| **CVD** | 1.82 | 0.56 | 0.08 |
| **All-cause death** | -0.34 | 0.29 | 0.29 |
| **NT-proBNP level** | -1.94 | 0.54 | 0.06 |
| **BNP level** | -0.66 | - | - |
| **6MWD** | 7.21 | 1.06 | 0.09 |
| **KCCQ-TSS** | -2.37 | - | - |
| **Adverse events** | -0.69 | 0.43 | 0.17 |

**Notes:** HHF: hospitalization for heart failure; CVD: cardiovascular death; BNP: B-type natriuretic peptide; NT-proBNP: N-terminal pro-B-type natriuretic peptide; 6MWD: 6 min-walk distance; KCCQ-TSS: the Kansas City Cardiomyopathy Questionnaire Total Symptom Score.
